# Retinoids Promote Mouse Bone Marrow-Derived Macrophage Differentiation and Efferocytosis via Upregulating Bone Morphogenetic Protein-2 and Smad3

**DOI:** 10.3390/cells11182928

**Published:** 2022-09-19

**Authors:** Éva Fige, Zsolt Sarang, László Sós, Zsuzsa Szondy

**Affiliations:** 1 Doctoral School of Dental Sciences, Faculty of Dentistry, University of Debrecen, 4032 Debrecen, Hungary; 2 Department of Biochemistry and Molecular Biology, Faculty of Medicine, University of Debrecen, 4032 Debrecen, Hungary; 3 Section of Dental Biochemistry, Department of Basic Medical Sciences, Faculty of Dentistry, University of Debrecen, 4032 Debrecen, Hungary

**Keywords:** macrophage differentiation, BMP-2, Smad3, efferocytosis, retinoids, inflammation

## Abstract

Clearance of apoptotic cells by bone marrow-derived macrophages differentiated from monocytes plays a central role in the resolution of inflammation, as the conversion of pro-inflammatory M1 macrophages to M2 macrophages that mediate the resolution process occurs during efferocytosis. Thus, proper efferocytosis is a prerequisite for proper resolution of inflammation, and failure in efferocytosis is associated with the development of chronic inflammatory diseases. Previous studies from our laboratory have shown that (13*R*)-all-*trans*-13,14-dihydroretinol (DHR), the product of retinol saturase, acting from day 4 of monocyte differentiation enhances the efferocytosis capacity of the resulted macrophages. Loss of retinol saturase in mice leads to impaired efferocytosis, and to development of autoimmunity. In the present paper, we report that in differentiating monocytes DHR, retinol, and all-*trans* retinoic acid all act directly on retinoic acid receptors and enhance the clearance of apoptotic cells by upregulating the expression of several efferocytosis-related genes. The effect of retinoids seems to be mediated by bone morphogenetic protein (BMP)-2, and the Smad3 transcription factor. In addition, retinoids also upregulate the expression of the vitamin D receptor and that of vascular endothelial growth factor A, indicating that altogether retinoids promote the generation of a pro-reparative M2 macrophage population during monocyte differentiation.

## 1. Introduction

Clearance of apoptotic cells by macrophages (efferocytosis) plays a crucial role in maintaining tissue homeostasis and in initiating the resolution of inflammation and tissue repair in case of infection or tissue injury. Macrophages normally take up dying cells by simultaneously using several phagocytic receptors. These receptors recognize cell surface changes on the apoptotic cells through either direct apoptotic cell–phagocyte interactions or serum opsonizing proteins that bridge apoptotic cell ligands and the efferocytic receptors [1]. The appearance of the phosphatidylserine (PS) on the surface of the apoptotic cell is the most ubiquitous indicator of dead cells [2]. It is directly recognized by BAI1 [3], stabilin-2 [4], and Tim-4 [5], while other phagocytic receptors utilize the bridging molecule milk fat globule EGF-factor 8 (MFG-E8) [6], thrombospondin-1 (THBS-1) [7], Gas6, protein S [8], or complement 1q (C1q) [9] for PS binding. Some efferocytic receptors, such as Tim-4 [10] or CD14 [11], facilitate tethering. In contrast, other receptors, such as CD36 [12], Mer tyrosine kinase (Mertk) [13], stabilin-2 [4], BAI1 [3], or integrin β3 with its coreceptor transglutaminase 2 (TG2) [14] trigger two evolutionally conserved parallel signaling pathways that initiate cytoskeletal reorganization via activating the small G protein Rac1 [15]. In addition, several scavenger receptors, such as the macrophage receptor with collagenous structure (MARCO) [16], have also been shown to contribute to efficient efferocytosis. Recognition of PS by MARCO has not yet been investigated but it is known to recognize ligands that are often polyanionic in nature, and was reported to also participate in the uptake of PS-positive exosomes [17]. Though in case of tissue damage or infection, tissue-resident macrophages participate in the initial sensing of danger, infiltrating macrophages differentiated from monocytes play the key role in removing dead cells [18]. Efficient efferocytosis induces an M1/M2 phenotypic change in these M1 pro-inflammatory macrophages leading to the formation of M2 macrophages that drive the downregulation of inflammation and also the tissue regeneration [19]. Thus, proper efferocytosis is essential for mediating the resolution of inflammation, as well as for initiating tissue repair. Failure in these processes leads to chronic inflammatory processes and to development of autoimmunity [1].

Previous studies in our laboratory have shown that the mRNA expression of retinol saturase [20], an enzyme that catalyzes a stereospecific saturation of the C13−C14 double bond of all-*trans*-retinol (ROL, vitamin A) to generate (13*R*)-all-*trans*-13,14-dihydroretinol (DHR), is induced during monocyte/macrophage differentiation [21]. In addition, its product DHR added during the generation of bone marrow-derived macrophages (BMDMs) upregulated TG2 during the last 2 days of the 5-day differentiation process and promoted efferocytosis by the resulted macrophages [21]. Impaired efferocytosis in retinol saturase null mice resulted in the development of a mild autoimmunity [21]. Though DHR is oxidized in vivo to all-*trans*-13,14-dihydroretinoic acid, a highly selective agonist of the retinoic acid receptor (RAR), and to 9-*cis*-13,14-dihydroretinoic acid, a highly selective agonist of the retinoid X (RXR) receptor [22], we could not detect the induction of other retinoid-sensitive efferocytosis-related genes by DHR [21] that we identified in our earlier studies in mature BMDMs [23]. Since previous reports indicated that the retinol saturase pathway might function independently of RAR receptors by regulating the activity of peroxisome proliferator-activated receptor (PPAR)γ [24] or that of carbohydrate response element binding protein (ChREBP) [25], the aim of the studies reported in this paper was to identify how DHR promotes efferocytosis administered during monocyte/macrophage differentiation.

## 2. Materials and Methods

### 2.1. Reagents 

All reagents were obtained from Sigma-Aldrich (Budapest, Hungary) except when indicated otherwise.

### 2.2. Animals

All the experiments were carried out with BMDMs differentiated from the bone marrow of 2–5 month-old C57BL/6 mice. Mice were maintained under specific pathogen-free conditions in the Central Animal Facility, University of Debrecen. All animal experiments were approved by the Animal Care and Use Committee of the University of Debrecen (DEMÁB) with permission numbers 7/2016/DEMÁB and 7/2021/DEMÁB.

### 2.3. Bone Marrow-Derived Macrophage Cell Culture and Treatment

Mice were sacrificed by isoflurane overdose. Bone marrow progenitors were obtained from the femur of 2 to 3-month-old mice by lavage with sterile physiological saline. Cells were differentiated for 5 days in DMEM medium supplemented with 10% conditioned medium derived from L929 cells [26], as a source for macrophage colony-stimulating factor (M-CSF), 2 mM glutamine, 100 U/mL penicillin, and 100 μg/mL streptomycin at 37 °C in 5% CO_2_. Non-adherent cells were washed away on the third day, and the same culture medium described above was readded. In the experiments, 1 μM ROL, 1 μM DHR, and 30 nM all-*trans* retinoic acid (ATRA) or 50 ng/mL recombinant bone morphogenetic protein (rBMP)-2 (R&D Systems, Minneapolis, MN, USA) alone or together with 0.5 μM AGN194310 (Tocris Bioscience, Abingdon, UK), 0.3 μM LDN193189 (Tocris Bioscience, Abingdon, UK), 2.5 μM PD98059 (Merck, Kenilworth, NJ, USA), 10 μM SIS3 (Tocris Bioscience, Abingdon, UK), 10 μM disulfiram or 25 μM N,N-diethylaminobenzaldehyde (DEAB) were added to the culture at the start of day 4 of the differentiation, and the cells were collected either 2–6 h later for either total mRNA sequencing or qRT-PCR determinations, or for 48 h for total mRNA sequencing and for efferocytosis assays.

### 2.4. Generation of Apoptotic Thymocytes

Thymi were collected from 4-week-old C57BL/6 mice, thymocytes were isolated and cultured for 24 h (10^7^ cells/mL) in DMEM medium supplemented with 2 mM glutamine, 100 U/mL penicillin, and 100 μg/mL streptomycin in the absence of serum to generate apoptotic thymocytes. Approximately 80% of the resulting cells were Annexin V-positive [27]. The number of cells was determined by Bürker chamber cell counting.

### 2.5. mRNA Sequencing

To obtain global transcriptome data from differentiating monocytes in the presence and absence of 1 μM ROL or 1 μM DHR, high throughput mRNA sequencing analysis was performed on an Illumina sequencing platform. The quality of total RNA samples was checked on Agilent BioAnalyzer using a Eukaryotic Total RNA Nano Kit, according to the manufacturer’s protocol (Agilent, Santa Clara, CA). Samples with RNA integrity number (RIN) values >7 were accepted for the library preparation process. RNA-Seq libraries were prepared from total RNA using the TruSeq RNA Sample preparation kit (Illumina), according to the manufacturer’s protocol. Briefly, poly-A RNAs were captured by oligo-dT conjugated magnetic beads, then the eluted mRNAs were fragmented at 94-Celsius. The first cDNA strand was generated by random priming reverse transcription, and after the second strand synthesis step double-stranded cDNA was generated. After repairing ends, A-tailing and adapter ligation steps, adapter-ligated fragments were amplified in enrichment PCR, and finally, sequencing libraries were generated. Sequencing runs were executed on an Illumina HiSeq2500 instrument using single-end 50 bp sequencing in the Genomic Medicine and Bioinformatics Core Facility of the University of Debrecen.

### 2.6. Functional Analysis of Differentially Expressed Genes (DEGs)

In order to gain insight into the biological function of the given transcripts, we used the online webtool Search Tool for the Retrieval of Interacting Genes v11.5 (https://string-db.org/ (accessed on 9 August 2022)) to identify over-represented gene ontology biological processes among the DEGs. Statistically significant enrichment of genes within the GO category was determined using the Aggregate Fold Change test with Benjamini and Hochberg FDR multiple test correction (FDR < 0.05) [28].

### 2.7. Quantitative Real-Time Polymerase Chain Reaction (qRT-PCR) Analysis of mRNA Expressions

Total RNA was isolated from BMDMs differentiated alone or exposed to retinoids and/or other compounds indicated in the figures at the start of day 4 of differentiation by using the TRI reagent, according to the manufacturer’s guidelines (ThermoFisher, Waltham, MA, USA). Total RNA was reverse transcribed into cDNA using a High Capacity cDNA Reverse Transcription Kit (Life Technologies, Budapest, Hungary) according to the manufacturer’s instruction. qRT-PCR was carried out in triplicate using pre-designed FAM-labeled MGB assays (Life Technologies, Budapest, Hungary) including LightCycler 480 Multiwell 384 white plates sealed with adhesive tapes on a Roche LightCycler LC 480 real-time PCR instrument. Relative mRNA levels were calculated using the comparative CT method and were normalized to β-actin mRNA. Catalog number of the TaqMan assays used for β-actin, Tgm2, THBS-1, Axl, CD36, Stab2, Tim4, CD14, Bmp2, Smad3, VEGFA, and RARβ were Mm02619580_g1, Mm00436979_m1, Mm00449032_g1, Mm00437221_m1, Mm00432403_m1, Mm00454684_m1, Mm00724709_m1, Mm00438094_g1, Mm01340178_m1, Mm01170760_m1, Mm00437306_m1, and Mm01319677_m1, respectively.

### 2.8. Efferocytosis Assays

Apoptotic thymocytes were stained with 2.5 µM DeepRed dye (Invitrogen, Carlsbad, CA, USA) for 24 h, and were added to 5-day-differentiated macrophages (2 × 10^5^) kept in 2 mL DMEM medium (supplemented with 10% L929 fibroblast-derived medium, 2 mM glutamine,100 U/mL penicillin, and 100 μg/mL streptomycin at 37 °C in 5% CO_2_) on 12 well TPP cell culture plates (Cat. number 92012) in 1:5 macrophage:target cell ratio. In some experiments LDN193189 and SIS3 were added together with the target cells. After coculture for 1 h, apoptotic cells were washed away. Macrophages were then detached by trypsinization and their fluorescence was analyzed using FACSCalibur. Macrophages were gated according to their forward and side scatter properties. Engulfing macrophages were identified within the macrophage population based on their high fluorescent emission detected in the FL4 channel.

### 2.9. Fluorescent Microscopy

Apoptotic thymocytes were stained with 2.5 µM DeepRed dye (Invitrogen, Carlsbad, CA, USA) for 24 h according to the protocol provided by the manufacturer, while BMDMs were stained with 10μM carboxyfluorescein diacetate succinimidyl ester (CFDA-SE; Thermo Fisher Scientific, Waltham, MA, USA). Apoptotic thymocytes were added to 2 × 10^5^ C57BL/6 macrophages in 1:5 macrophage:target cell ratio for 1 h, then the remaining cells were washed away. BMDMs were then fixed with 1% paraformaldehyde. Pictures were then taken on a fluorescent microscope (FLoid™ Cell Imaging Station, ThermoFisher, Waltham, MA, USA.

### 2.10. Statistical Analysis

All the data are representative of at least three independent experiments carried out with macrophages isolated from three different mice. Values are expressed as mean ± S.D. For differences between two groups, two-tailed unpaired Student’s t-test, for comparisons between multiple groups one-way ANOVA (with Tukey’s multiple comparisons test) were used. All statistical analyses were performed using GraphPad Prism 6.01 and a *p* value < 0.05 was considered as significant and is indicated by asterisk (*).

## 3. Results

### 3.1. Dihydroretinol Administered during Monocyte Differentiation Enhances Efferocytosis of Macrophages by Upregulating the Expression of Several Efferocytosis-Related Molecules

**Figure 1:**
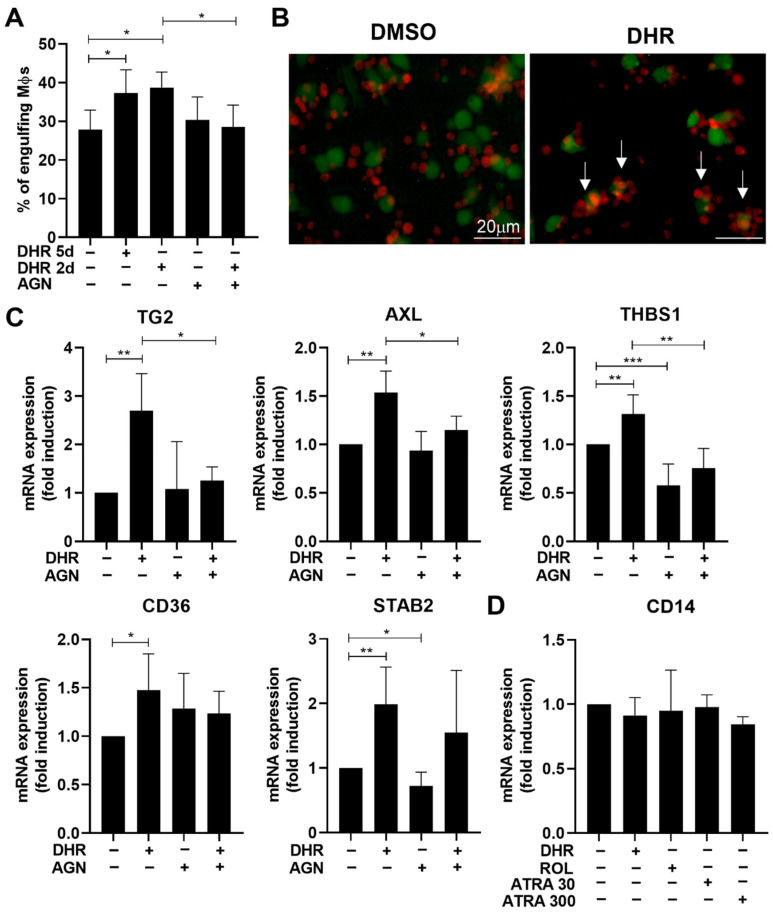
Dihydroretinol administered during monocyte differentiation enhances efferocytosis of macrophages by upregulating the expression of several efferocytosis-related molecules in an RAR-dependent manner. (**A**) Percentage of engulfing macrophages differentiated in the absence or presence of 1 μM DHR administered either during the whole 5 days or only the last 2 days of their differentiation in the presence or absence of 0.5 μM AGN194310, a highly selective pan-RAR antagonist. (**B**) Representative fluorescent microscopic images of macrophages (green) engulfing apoptotic thymocytes (red) after being kept in the presence and absence of 1 μM DHR during the last 2 days of differentiation. Non-engulfed apoptotic cells appear as red, while the engulfed appear as orange colors, as a result of the overlapping green and red colors of the two types of cells. Arrows point to macrophages covered by tethering apoptotic thymocytes. (**C**) mRNA expression of several efferocytosis-related genes in macrophages exposed to 1 μM DHR during the last 2 days of their differentiation in the presence or absence of 0.5 μM AGN194310 determined by qRT-PCR. β-actin was used as a reference gene. (**D**) Lack of induction of CD14 mRNA expression by 1 μM DHR, 1 μM ROL, and by 30 or 300 nM ATRA. All data are expressed as mean ± SD (*n* = 3 in **A**, and *n* = 4 in **C**)). Asterisks indicate statistically significant difference (* *p* < 0.05, ** *p* < 0.01, and *** *p* < 0.001). Each culture contained 0.5% *v*/*v* DMSO.

To confirm our previous results [21], we tested whether addition of DHR during monocyte differentiation indeed affects efferocytosis of the resulting macrophages. As seen in Figure 1A, DHR significantly enhanced the phagocytic capacity of macrophages tested after 5 days of differentiation. Since our previous studies indicated that DHR upregulates TG2 and MFG-E8 only during the last 2 days of differentiation [21], we also tested whether DHR is able to enhance efferocytosis if it is only added from the start of day 4 of differentiation. As seen in Figure 1A, DHR was able to induce the same increase in the efferocytic capacity as if it was present during the whole 5 day-period of differentiation, indicating that DHR acts only during the last two days to enhance efferocytosis. Fluorescent images of engulfing macrophages revealed that exposure to DHR not only enhanced the phagocytic uptake of apoptotic cells by macrophages but also promoted the tethering of apoptotic cells to them (Figure 1B).

To identify which efferocytosis-related genes are upregulated by the end of differentiation, DHR was added to differentiating monocytes from the start of day 4, and the differentiated macrophages were collected 2 days later for total mRNA sequencing. From the collected cells we identified 781 DEGs between DHR-treated and non-treated BMDMs (based on at least 1.5-fold change and corrected *p* value < 0.05). A total of 357 transcripts showed an increase (Table S1), and 424 transcripts showed decreased gene expression (Table S2) in the DHR-treated cells. The mean FC of decreased and increased transcripts was −2.66 ± 1.89 and 6.55 ± 22.52, respectively. The median FC value of decreased and increased transcripts was −2.08 and 2.36, respectively. Functional analysis revealed that genes related to monocyte differentiation, phagocytosis, and wound healing response are overrepresented among the upregulated DEGs (Table S3). Among the genes showing enhanced expression in DHR-treated macrophages, we found 10 related to phagocytosis of apoptotic cells (Table 1), though only 5 were listed by the Gene Ontology record.

Five of these were efferocytosis receptors or coreceptors that included stabilin-2, TG2, Axl, MARCO, and CD36, one was a bridging molecule (THBS-1), and one was a Rab family member (Rab20) contributing to phagosome maturation [29]. In line with our previous findings [21], besides TG2 and THBS-1 the expression of the other retinoid-sensitive phagocytosis genes that we identified in mature BMDMs (Tim-4, C1q, CD14) [23] were not affected by DHR administered during monocyte differentiation. In addition, we detected a very strong induction of the calmodulin-dependent kinase 1 (Camkk1) enzyme and that of the purinergic receptor P2X (P2X_1_). Camkk1 was shown to be upregulated during macrophage differentiation [30] and together with Camkk2 to positively regulate AMP-dependent kinase activity [31] known to enhance efferocytosis [32]. P2X_1_, on the other hand, was found to promote the tethering of apoptotic cells to macrophages in the presence of ATP [33]. In addition, we found the induction of Smad3, a transcription factor known to enhance efferocytosis by acting directly during efferocytosis, as well as by enhancing the expression of several efferocytosis-related genes [34]. Interestingly, this screen did not pick up the bridging molecule MFG-E8 that we found in our previous study, which was also DHR-sensitive [21]. Upregulation of three tethering receptors (CD14, Tim-4, and P2X_1_) might explain the observed increase in the tethering of apoptotic cells following DHR exposure of macrophages during their differentiation (Figure 1B).

To verify the findings, we determined the mRNA expression of five DHR-induced efferocytosis-related molecules in BMDMs by qRT-PCR and found their increased expressions (Figure 1C) as compared to the non-treated. The expression of Tim-4, C1q, or CD14, three previously identified retinoic acid-sensitive phagocytosis receptors [23], however, was not induced (CD14 is shown in Figure 1D), even if the ATRA concentration was raised to 300 nM.

### 3.2. DHR Administered during Monocyte Differentiation Promotes Efferocytosis via Directly Affecting RARs

Since previous studies indicated that DHR could mediate its effects via being converted to dihydroretinoic acids that activate RARs [22], we tested whether ROL that can be converted to ATRA [35] or ATRA that is the primary ligand of RARs could replace DHR in regulating efferocytosis at the end of the 5 day-monocyte differentiation, if added at the start of day 4 of differentiation.

**Figure 2:**
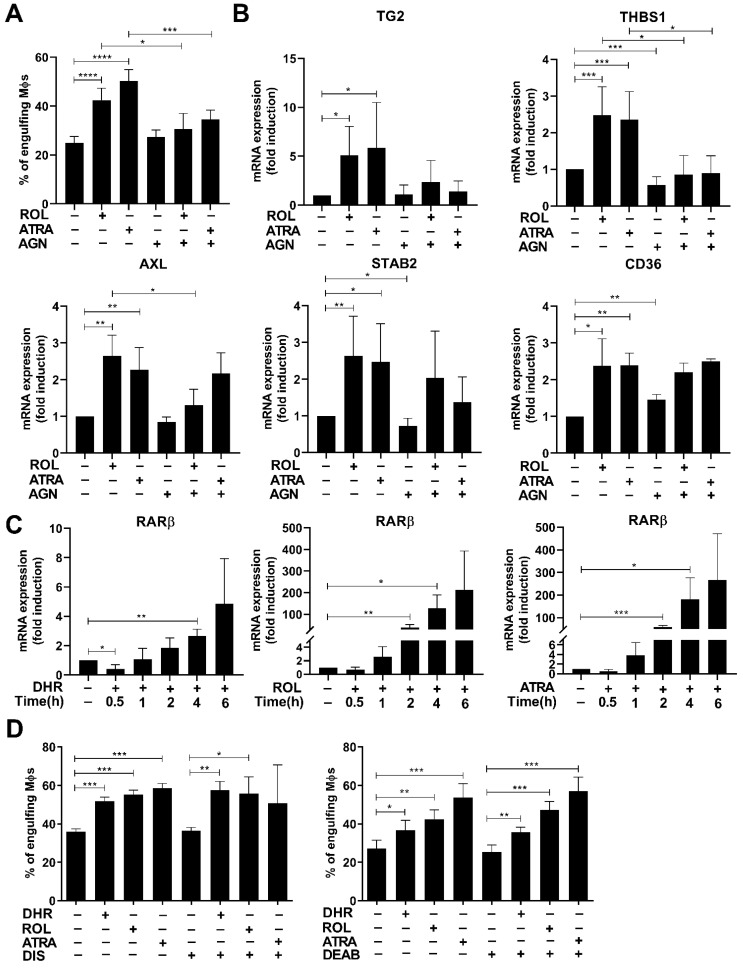
Both retinol and all-*trans* retinoic acid administered during the last two days of macrophage differentiation enhances efferocytosis of the resulting BMDMs by upregulating the expression of several efferocytosis-related molecules in a RAR-dependent manner. (**A**) Percentage of engulfing macrophages differentiated in the absence or presence of 1 μM ROL or 30 nM ATRA alone or in the presence of 0.5 μM AGN194310 administered during the last 2 days of their differentiation. (**B**) mRNA expression of several efferocytosis-related genes in macrophages exposed to 1 μM ROL or 30 nM ATRA alone or in the presence of 0.5 μM AGN194310 during the last 2 days of their differentiation determined by qRT-PCR. (**C**) Time-dependent induction of the RARβ mRNA expression by 1 μM DHR, 1 μM ROL, and 30 nM ATRA added at day 4 to differentiating macrophages. (**D**) Neither the aldehyde dehydrogenase inhibitor disulfiram (10 μM) nor the retinaldehyde dehydrogenase inhibitor DEAB (25 μM) affects the retinoid-induced efferocytosis of BMDMs. Each compound was administered from day 4 of macrophage differentiation. All data are expressed as mean ± SD (*n* = 3). β-actin was used as a reference gene for all qRT-PCR determinations. Asterisks indicate statistically significant difference (* *p* < 0.05, ** *p* < 0.01, and *** *p* < 0.001, and **** *p* < 0.0001). Each culture contained 0.5% *v*/*v* DMSO.

As seen in Figure 2A, both ROL and ATRA added at the start of day 4 during macrophage differentiation enhanced phagocytosis of apoptotic thymocytes, ATRA being more effective. ROL and ATRA were also capable of inducing the same 5 efferocytosis-related genes tested 48 h later, which were induced by DHR (Figure 2B). In addition, AGN194310, a highly selective pan-RAR antagonist, attenuated the DHR-induced enhancement both in the efferocytic capacity of macrophages (Figure 1A), and in the expressions of several efferocytosis-related genes (Figure 1C), similar to its inhibition of those induced by ROL and ATRA (Figure 2A,B). Interestingly, however, not all the retinoid-induced expression of phagocytosis-related genes was prevented by administration of the pan-RAR transcription antagonist compound indicating that only the expression of TG2 and THBS-1 was induced clearly in an RAR transcription-dependent manner. Interestingly, these were the two phagocytic molecules that we previously identified to be induced in an RAR-dependent manner in mature BMDMs as well [23]. AXL, stabilin-2 or CD36, however, seem to be induced by a different mechanism. This mechanism might involve direct interaction of RARs with other transcription factors leading to their transrepression or transactivation with which a transcription antagonist does not necessarily interfere [36,37]. The RAR involved is very likely RARβ, since it was the only RAR induced by DHR (Table S1), and we could confirm its induction by all of the three retinoids detected by qRT-PCR as well (Figure 2C). In addition, these data also demonstrate that different genes are open for RAR-mediated regulation at the start of day 4 of differentiation, compared to the differentiated macrophages.

To convert ROL or DHR to the relevant retinoic acid (ATRA or all-*trans* dihydroretinoic acid, respectively), the subsequent action of the same aldehyde dehydrogenases and retinaldehyde dehydrogenases is required [20,22,35]. Interestingly, however, neither the aldehyde dehydrogenase inhibitor disulfiram, which inhibits the first step, nor the retinaldehyde dehydrogenase inhibitor DEAB, which inhibits the second step of this conversion, prevented the ROL- or the DHR-induced efferocytosis of differentiating macrophages (Figure 2D). These data indicate that ROL or DHR might regulate RAR activity by direct binding to the receptor [38], rather than being converted to retinoic acid. As expected, these compounds had no effect on the ATRA-induced efferocytosis either.

### 3.3. Retinoids Added from Day 4 of Monocyte Differentiation Induce the Expression of Bone Morphogenetic Protein 2 (BMP-2)

To identify which genes might mediate the effect of retinoids on efferocytosis added on day 4 of differentiation, DHR and ROL were added to differentiating macrophages at the start of day 4, and cells were collected 2 h later for total mRNA sequencing.

As seen in Table 2, we identified 106 DEGs between DHR-treated and non-treated differentiating macrophages (based on at least 1.5-fold change and corrected *p* value < 0.05). A total of 74 transcripts showed an increase, and 32 transcripts showed decreased gene expression in the DHR-treated cells. The mean FC of decreased and increased transcripts was −2.28 ± 0.72 and 3.34 ±4.4, respectively. The median FC value of decreased and increased transcripts was −2.08 and 2.15, respectively. The same genes were induced by ROL as well (Table S4), indicating that DHR and ROL affect the same transcriptional pathways. From the upregulated genes, we selected the BMP-2 growth factor as a candidate gene because it was found to be strongly upregulated in the 48 h samples as well (Table S1), and it was also shown previously to be a RAR target gene [39].

**Figure 3:**
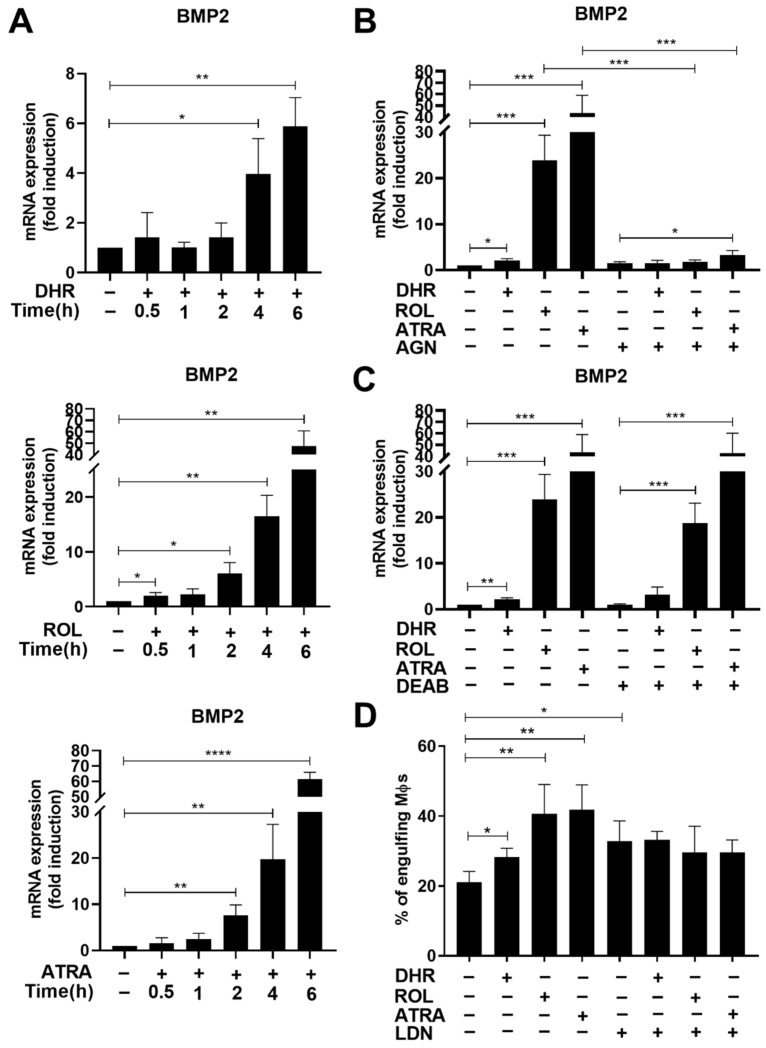
BMP-2 is induced by retinoids during macrophage differentiation and contributes to the retinoid-enhanced efferocytosis. (**A**) Time-dependent increase in the BMP-2 mRNA expression of differentiating macrophages exposed to 1 μM DHR, 1 μM ROL, or 30 nM ATRA at day 4 of differentiation. (**B**) Inhibition of retinoid-induced BMP-2 mRNA expression by AGN194310 (0.5 μM) detected at 6 h after retinoid administration in day 4 differentiated macrophages. (**C**) Inhibition of retinaldehyde dehydrogenases by DEAB (25 μM) has no effect on the retinoid-induced BMP-2 mRNA expression detected at 6 h after retinoid administration in day 4 differentiated macrophages. (**D**) LDN193189 (0.3 μM), a BMP inhibitor, added together with the retinoids from day 4 of macrophage differentiation attenuates the induction of efferocytosis by retinoids detected 2 days later. All data are expressed as mean ± SD (*n* = 4). Β-actin was used as a reference gene for all qRT-PCR determinations. Asterisks indicate statistically significant difference (* *p* < 0.05, ** *p* < 0.01, and *** *p* < 0.001, and **** *p* < 0.0001). Each culture contained 0.5% *v*/*v* DMSO.

Indeed, the mRNA expression of BMP-2 was induced in a time-dependent manner by all the three retinoids added on day 4 of differentiation (Figure 3A). The addition of AGN194310 completely prevented the induction of BMP-2 mRNA by all the three retinoids tested 6 h after retinoid addition (Figure 3B), proving the transcriptional involvement of RARs. Since inhibition of retinaldehyde dehydrogenases by DEAB had no effect on the retinoid-induced induction of efferocytosis (Figure 2D), we tested whether retinoid-induced induction of BMP-2 expression is affected by it. However, as seen in Figure 3C, DEAB did not affect the BMP-2 mRNA induction either, indicating again that ROL and DHR might regulate RAR activity by direct binding.

### 3.4. BMP-2 Contributes to the Retinoid-Induced Efferocytic Capacity in Differentiating Macrophages

To test the potential involvement of BMP-2 in the retinoid-induced efferocytosis, retinoids were added together with the BMP receptor (ALK2/3 serine/threonine kinase) inhibitor LDN193189. As seen in Figure 3D, though LDN193189 added alone during macrophage differentiation increased the basal efferocytosis by the resulted BMDMs, the retinoid-induced enhancement in efferocytosis was significantly inhibited by it. If the BMP-2 receptor inhibitor was added to non-treated BMDMs at the start of efferocytosis, it did not affect the 1 h efferocytosis (data not shown) indicating that the BMP-2 receptor signaling must act during the macrophage differentiation prior to efferocytosis.

### 3.5. Smad3 Is also Induced by Retinoids

**Figure 4:**
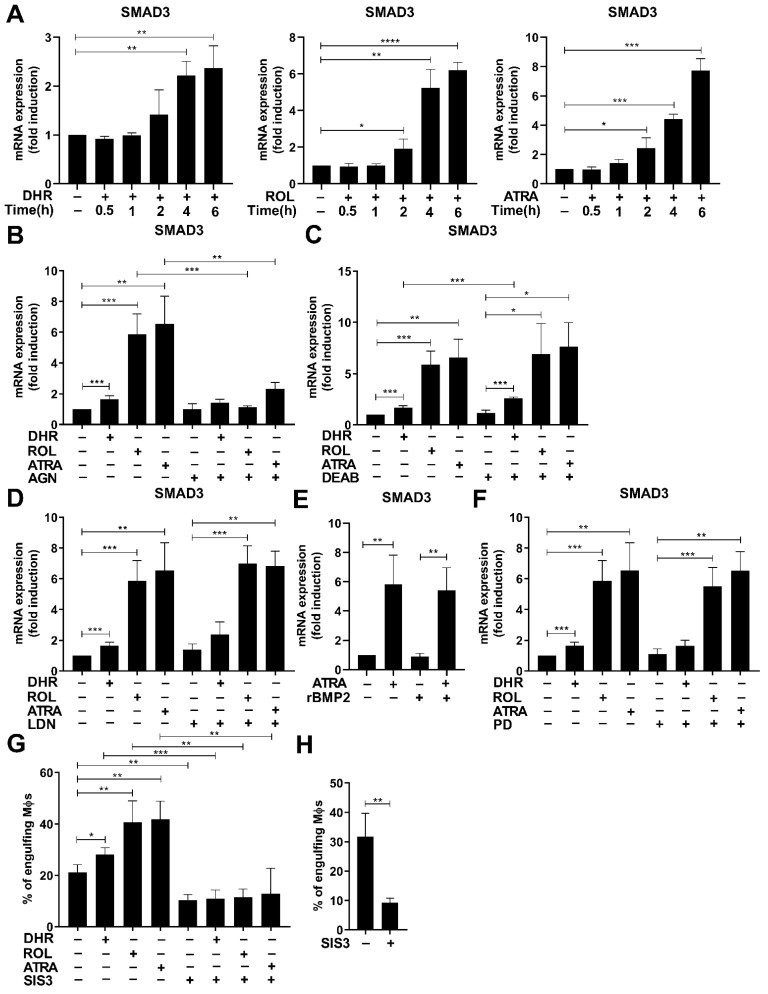
Smad3 is induced by retinoids during macrophage differentiation and contributes to retinoid-enhanced efferocytosis. (**A**) Time-dependent increase in the Smad3 mRNA expression of differentiating macrophages exposed to 1 μM DHR, 1 μM ROL, or 30 nM ATRA at day 4 of differentiation. (**B**) Inhibition of retinoid-induced Smad3 mRNA expression by AGN194310 (0.5 μM) detected at 6 h after retinoid administration in 4-day differentiated macrophages. (**C**) Inhibition by retinaldehyde dehydrogenases by DEAB (25 μM) has no effect on the retinoid-induced Smad3 mRNA expression detected at 6 h after retinoid administration in day 4 differentiated macrophages. (**D**) LDN193189 (0.3 μM), a BMP inhibitor, added together with the retinoids on day 4 of macrophage differentiation does not affect the retinoid-induced Smad3 expression detected at 6 h after retinoid administration. (**E**) Recombinant BMP-2 does not affect the retinoid-induced expression of Smad3 in 4-day differentiated macrophages detected 6 h after retinoid administration. (**F**) PD98059 (5 μM), a MEK1 inhibitor, added together with the retinoids on day 4 of macrophage differentiation, has no effect on retinoid-induced Smad3 expression. (**G**) Sis3 (0.5 μM), added together with the retinoids on day 4 of macrophage differentiation attenuates the induction of efferocytosis by retinoids detected 2 days later. (**H**) Sis3 (0.5 μM) added together with apoptotic thymocytes significantly attenuates efferocytosis by BMDMs. All data are expressed as mean ± SD (*n* = 4). β-actin was used as a reference gene for all qRT-PCR determinations. Asterisks indicate statistically significant difference (* *p* < 0.05, ** *p* < 0.01, and *** *p* < 0.001, and **** *p* < 0.0001). Each culture contained 0.5% *v*/*v* DMSO.

In addition to BMP-2, following DHR administration we also observed the mRNA induction of Smad3 (Table 2), a transcription factor known to mediate the effect of transforming growth factor (TGF)-β and known to be associated in macrophages with efferocytosis [34]. Indeed, the mRNA expression of Smad3 was induced in a time-dependent manner by all three retinoids added on day 4 of differentiation (Figure 4A). The addition of AGN194310 completely prevented the induction of Smad3 mRNA by all the three retinoids tested 6 h after retinoid addition (Figure 4B), proving the transcriptional involvement of RARs. Inhibition of retinaldehyde dehydrogenases by DEAB, on the other hand, as in the case of efferocytosis or that of the mRNA expression of BMP-2, had no effect on its retinoid-induced induction (Figure 4C).

Next, we tested whether the induction of Smad3 mRNA by retinoids involves BMP-2. However, as shown in Figure 4D, neither was its induction by retinoids affected by the BMP-2 receptor inhibitor LDN193189 nor was it induced by recombinant BMP-2 added alone or together with ATRA (Figure 4E).

Previous studies indicated that TGF-β is produced and signals in an autocrine manner by differentiating monocytes [40], and TGF-β can upregulate Smad3 expression via mitogen-activated protein kinase kinase-1 (MEK1) [41]. However, inhibition of MEK1 by PD98059 (Figure 4F) had no effect on the retinoid-induced Smad3 expression. These data indicate that neither BMP-2 nor TGF-β1 mediates the effect of retinoids on the expression of Smad3.

### 3.6. Smad3 also Contributes to the Retinoid-Induced Efferocytosis during Monocyte Differentiation

To test whether the Smad3 transcription factor is involved in retinoid-induced efferocytosis during macrophage differentiation, at day 4 of differentiation retinoids were added together with SIS3, a TGFβ-1 receptor (ALK5 kinase) inhibitor known to interfere with Smad3 signaling, and the efferocytosis capacity of the cells was determined 2 days later. As shown in Figure 4G, SIS3 significantly inhibited basal efferocytosis, and prevented the induction of efferocytosis by retinoids implying a determining role of TGFβ receptor/Smad3 signaling in the upregulation of efferocytosis during macrophage differentiation. In addition, it inhibited efferocytosis of fully maturated BMDMs added at the start of efferocytosis to a similar degree (Figure 4H), indicating a determining role of Smad3 during the efferocytosis process itself. In accordance with our finding, direct involvement of Smad3 in the efferocytosis process has been previously observed [34]. However, we have to note that exposure to SIS3 for 2 days significantly reduced (by 45% as compared to controls, data not shown) the number of viable macrophages in the 5-day cultures, in accordance with the observation that TGF-β1, which signals in an autocrine manner in differentiating monocytes strongly contributes to their survival [40].

### 3.7. Retinoids also Upregulate the Expression of Vascular Endothelial Growth Factor (VEGF) A

Among the early upregulated genes (Table 2), in addition to those that mediate the effect of retinoids on efferocytosis, we also found genes, such as vitamin D receptor or vascular endothelial growth factor (VEGF) A, which are associated with the M2 polarization of monocytes/macrophages. Vitamin D receptor is known to promote M2 conversion of macrophages [42], while VEGFA is a growth factor known to be produced by M2 pro-reparative monocytes/macrophages that drives angiogenesis [43]. Thus, we also looked at the regulation of VEGFA mRNA expression.

**Figure 5:**
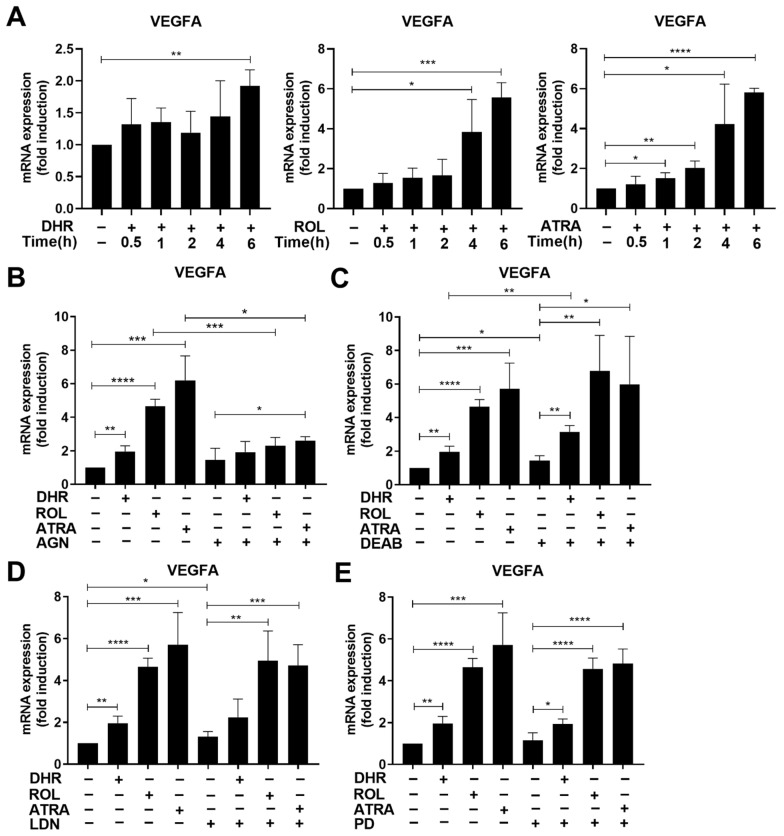
VEGFA is also induced by retinoids during macrophage differentiation. (**A**) Time-dependent increase in the VEGFA mRNA expression of differentiating macrophages exposed to 1 μM DHR, 1 μM ROL or 30 nM ATRA at day 4 of differentiation. (**B**) Inhibition of retinoid-induced VEGFA mRNA expression by AGN194310 (0.5 μM) detected at 6 h after retinoid administration in 4-day differentiated macrophages. (**C**) Inhibition by retinaldehyde dehydrogenases by DEAB (25 μM) has no effect on the retinoid-induced VEGFA mRNA expression detected at 6 h after retinoid administration in day 4 differentiated macrophages. Neither (**D**) LDN193189 (0.3 μM), nor (**E**) PD98059 (5 μM), a MEK1 inhibitor, added together with the retinoids on day 4 of macrophage differentiation, have an effect on retinoid-induced VEGFA mRNA expression detected 6 h later. All data are expressed as mean ± SD (*n* = 4). β-actin was used as a reference gene for all qRT-PCR determinations. Asterisks indicate statistically significant difference (* *p* < 0.05, ** *p* < 0.01, and *** *p* < 0.001, and **** *p* < 0.0001). Each culture contained 0.5% *v*/*v* DMSO.

Similar to BMP-2 and Smad3, retinoids induced the mRNA expression of VEGFA in a time- and RAR transcription-dependent manner (Figure 5A,B), and DEAB had no effect on this induction either (Figure 5C). Lack of effect of LDN193189 or PD98059 (Figure 5D,E) indicated that ALK2-, ALK3-, or MEK1-mediated signaling are not involved in the regulation of VEGFA mRNA expression. However, previous studies using Smad3 null macrophages demonstrated the involvement of Smad3 in the regulation of VEGFA in macrophages [34], indicating that retinoids might indirectly increase its expression via elevating Smad3 levels (Figure 4A).

Previous studies indicated that compared to in vivo differentiated peritoneal macrophages, BMDMs lie further toward the M1 end of the M1–M2 polarization spectrum [44]. Interestingly, BMP-2 itself was shown to be produced by M2 macrophages [45,46]. Thus, we searched for M2 marker genes among those upregulated by the last 48 h DHR treatment during macrophage differentiation to determine whether retinoid-treatment might promote M2 polarization. As seen in Table 3, DHR-exposure upregulated the expression of several M2-associated macrophage genes.

## 4. Discussion

Previous studies from our laboratory have indicated that DHR acting during the last 2 days of mouse bone marrow-derived macrophage differentiation enhances the phagocytic capacity of the resulting BMDMs [21], but it affects a different set of efferocytosis genes as compared to retinoic acid-exposed BMDMs [23]. That is why we concluded that DHR might act via a different signaling pathway in differentiating macrophages than retinoic acid does in mature BMDMs in the promotion of efferocytosis. The data presented in this paper, however, reveal that DHR acts on the same signaling pathway as ROL and ATRA mediated by RARs, but a different set of genes is sensitive to retinoid treatment in differentiating as compared to the differentiated macrophages. Interestingly, however, inhibitors of the conversion of ROL or DHR to retinoic acids did not prevent their effect on gene expression or on efferocytosis, indicating that ROL and DHR might directly activate RARs in differentiating macrophages without being converted to the relevant retinoic acids [38]. Our data also reveal that not all effects of retinoids on the phagocytosis receptor gene expressions were mediated by the direct transcriptional activity of RARs. It is well known that in addition to promoting transcription, ATRA-bound RARs can mediate AP1 transrepression [36], and we ourselves have shown that RARs are also capable of promoting the transcriptional activity of the glucocorticoid receptor acting via direct receptor/receptor interaction without the need for DNA binding [37]. Certain synthetic retinoids can differentiate between these biological activities because different parts of the ATRA-bound receptor participate in these activities, and synthetic retinoids do not necessarily compete with all of them [36,37]. In addition, retinoid-bound RARs might also have non-genomic effects [47], which indirectly affect transcription.

We identified two mediators of retinoid signaling in monocytes that contribute to the observed increase in efferocytosis capacity: BMP-2 and Smad3. Both BMP-2 and Smad3 have already been shown to be direct RAR-regulated target genes [40,48], and according to our data neither the retinoid-induced BMP-2 nor the differentiation-related TGF-β1 signaling contribute the upregulation of Smad3 by retinoids. BMP-2 generally signals via the Smad 1/5/8 and possibly 9 transcription factors [49]. In addition, however, in differentiating cells BMP-2 was found to signal also via a noncanonical pathway that involves BMP-2/TGF-β1 receptor heterodimers and Smad3 [50]. Though we have not investigated this possibility in detail, based on our results we cannot exclude that retinoids might promote efferocytosis not only via BMP-2 and TGF-β but also in a BMP-2/ BMP-2 and TGF-β1 heterodimer receptor/Smad3-dependent manner in differentiating macrophages.

Monocytes are bone marrow-derived innate immune cells that circulate for approximately 2 days before being cleared from circulation. Circulating monocytes scan for pathogens and respond to inflammatory signals needed to perform their phagocytic functions, or monocytes can tissue migrate to become macrophages. The resulting macrophages are not a uniform cell population; and there is significant macrophage heterogeneity determined by the immune environment [51]. In mice, monocyte derived macrophages are typically classified as either pro-inflammatory M1 (Ly6C^Hi^) or M2 (Ly6C^Lo^) pro-reparative cells generated following efferocytosis. The Ly6C^Lo^ macrophages can be divided into three further groups participating in the resolution of inflammation, antigen presentation, and tissue repair, and the later are known to release growth factors and pro-angiogenic and tissue repair factors, such as VEGFA, growth differentiation-factor 3 (GDF3), or insulin-like growth factor-1 (IGF-1) [51].

Monocyte differentiation to macrophages in vivo is triggered by migration across the endothelial barrier, which is constituted by both endothelial cells and their underlying basement membrane [52]. In case of BMDM differentiation, the monocyte and the macrophage differentiation stages are not so clearly separated. This difference is very likely related to the fact that BMDM differentiation is an adherence-mediated maturation, as inhibition of this adherence results in monocyte formation [53]. Thus, BMDM differentiation partially mimics the in vivo monocyte-derived macrophage differentiation process [44].

Bone marrow-derived monocytes very often appear and differentiate into macrophages in tissues where an increased rate of cell death occurs, such as during skeletal muscle injury [54] or in the case of high fat diet-induced death of adipocytes [55]. Thus, a retinoid-induced increase in their efferocytosis capacity will contribute to the efficient clearance of dead cells in these tissues to prevent chronic inflammation and the development of autoimmunity [56]. In addition, Smad3 is also a determinant mediator of the anti-inflammatory effects of TGF-β [57], known to be released by engulfing macrophages during efferocytosis [58].

Upregulation of the expression of vitamin D receptor or that of VEGFA by retinoids during monocyte differentiation also indicates that retinoids promote the generation of such M2-type macrophages that participate in the regulation of tissue repair. Upregulation of TG2, or ALDH1a2 detected 48 h after retinoid addition also supports this view [59]. Interestingly, the involvement of Smad3 in mediating tissue repair responses of macrophages was just reported in an infarcted myocardium model [60].

In line with our observations, M2 polarization of differentiating monocytes by ATRA released by a mouse tumor itself was observed in the environment of the tumor, where the resulting tumor-associated M2-like macrophages mediated immune-suppression and promoted tumor growth [61]. ATRA was also found to promote M2 polarization of differentiating monocytes during L. major infection in mice [62]. Altogether our data provide further proof that retinoids acting during macrophage differentiation promote the generation of M2-like, immune-suppressive, pro-reparative macrophages, and we demonstrate that the effects of retinoids are partially mediated via BMP-2 and Smad3.

## Figures and Tables

**Table 1:** List of differentially expressed efferocytosis-related genes between BMDMs treated with DMSO- or 1 μM DHR during the last 48 h of their differentiation (based on at least 1.5-fold change and corrected *p* value < 0.05).

Corr. *p* Val.	FC	Gene Symbol	Gene Title
1.21 × 10^−6^	212.53	Camkk1	calcium/calmodulin-dependent protein kinase 1, alpha
1.73 × 10^−3^	8.35	P2rx1	purinergic receptor P2X, ligand-gated ion channel, 1
1.04 × 10^−7^	4.1	Smad3	SMAD family member 3
4.38 × 10^−3^	3.65	Marco	macrophage receptor with collagenous structure
1.34 × 10^−7^	3.59	Rab20	RAB20, member RAS oncogene family
1.07 × 10^−3^	3.21	Stab2	stabilin 2
4.88 × 10^−4^	1.99	THBS-1	thrombospondin 1
6.24 × 10^−8^	1.58	Tgm2	transglutaminase 2, C polypeptide
5.23 × 10^−3^	1.56	Axl	AXL receptor tyrosine kinase
1.43 × 10^−4^	1.53	CD36	CD36 antigen

**Table 2:** List of 106 differentially expressed transcripts between 2 h DMSO- and 2 h DHR (1 μM)-treated 4 days differentiated macrophages (based on at least 1.5-fold change and corrected *p* value < 0.05).

Upregulated Transcripts			
Corr. *p* Val.	FC	Gene Symbol	Gene Title
1.8 × 10^−8^	30.3	Hic1	hypermethylated in cancer 1
1.2 × 10^−6^	25.0	Camkk1	calcium/calmodulin-dependent protein kinase 1, alpha
2.4 × 10^−3^	10.8	Gm15927	predicted gene 15927
8.9 × 10^−8^	7.6	Bmp2	bone morphogenetic protein 2
2.1 × 10^−5^	7.4	B230378P21Rik	RIKEN cDNA B230378P21 gene
3.2 × 10^−6^	6.1	Art2a-ps	ADP-ribosyltransferase 2a, pseudogene
2.4 × 10^−3^	5.1	Dll1	delta-like 1 (Drosophila)
2.8 × 10^−5^	5.0	Tox3	TOX high mobility group box family member 3
1.6 × 10^−3^	4.8	Fam20a	family with sequence similarity 20, member A
4.3 × 10^−5^	4.5	Kcnip3	Kv channel interacting protein 3, calsenilin
6.1 × 10^−3^	4.2	AI848285	expressed sequence AI848285
3.4 × 10^−4^	4.1	Kcng1	potassium voltage-gated channel, subfamily G, member 1
2.9 × 10^−4^	3.9	Il2rb	interleukin 2 receptor, beta chain
1.5 × 10^−2^	3.3	Vash1	vasohibin 1
2.0 × 10^−7^	3.2	Gm13431	predicted gene 13431
5.4 × 10^−3^	3.1	Bfsp1	beaded filament structural protein 1, in lens-CP94
8.6 × 10^−5^	3.1	Hbegf	heparin-binding EGF-like growth factor
4.9 × 10^−8^	3.1	Pram1	PML-RAR alpha-regulated adaptor molecule 1
9.6 × 10^−3^	2.9	Fam124a	family with sequence similarity 124, member A
5.9 × 10^−5^	2.8	Shcbp1l	Shc SH2-domain binding protein 1-like
1.2 × 10^−6^	2.8	Nppa	natriuretic peptide type A
2.1 × 10^−7^	2.8	Hivep2	human immunodeficiency virus type I enhancer binding protein 2
3.8 × 10^−5^	2.7	Robo3	roundabout homolog 3 (Drosophila)
9.1 × 10^−7^	2.7	Vegfa	vascular endothelial growth factor A
3.2 × 10^−3^	2.7	Gm9733	predicted gene 9733
1.0 × 10^−7^	2.7	Smad3	SMAD family member 3
8.3 × 10^−4^	2.6	Tubb3	tubulin, beta 3 class III
5.4 × 10^−3^	2.5	Gm7148	predicted gene 7148
1.7 × 10^−4^	2.3	Btnl4	butyrophilin-like 4
3.4 × 10^−3^	2.3	Rpsa-ps3	ribosomal protein SA, pseudogene 3
6.5 × 10^−8^	2.3	Osgin1	oxidative stress induced growth inhibitor 1
8.8 × 10^−9^	2.2	Dtx4	deltex 4 homolog (Drosophila)
1.3 × 10^−10^	2.2	Ptgs1	prostaglandin-endoperoxide synthase 1
1.3 × 10^−7^	2.2	Rab20	RAB20, member RAS oncogene family
4.8 × 10^−3^	2.2	Gm11870	predicted gene 11870
6.9 × 10^−7^	2.2	Il21r	interleukin 21 receptor
4.4 × 10^−4^	2.1	Map6d1	MAP6 domain containing 1
1.9 × 10^−3^	2.1	Corin	corin
2.6 × 10^−9^	2.1	2510009E07Rik	RIKEN cDNA 2510009E07 gene
2.0 × 10^−6^	2.1	Socs2	suppressor of cytokine signaling 2
1.1 × 10^−9^	2.0	Mcart1	mitochondrial carrier triple repeat 1
2.2 × 10^−7^	2.0	Asb10	ankyrin repeat and SOCS box-containing 10
6.9 × 10^−8^	2.0	Neurl3	neuralized homolog 3 homolog (Drosophila)
1.4 × 10^−4^	1.9	Mex3b	mex3 homolog B (C. elegans)
2.0 × 10^−5^	1.9	Hs3st3b1	heparan sulfate (glucosamine) 3-O-sulfotransferase 3B1
1.3 × 10^−3^	1.9	Gm15708	predicted gene 15708
1.1 × 10^−2^	1.9	Pih1d2	PIH1 domain containing 2
1.4 × 10^−5^	1.9	Hrh1	histamine receptor H1
4.5 × 10^−3^	1.9	Elmo3	engulfment and cell motility 3
1.3 × 10^−5^	1.9	AA467197	expressed sequence AA467197
2.5 × 10^−6^	1.8	Gm22	predicted gene 22
4.5 × 10^−8^	1.8	Fam117a	family with sequence similarity 117, member A
2.1 × 10^−6^	1.8	Dchs1	dachsous 1 (Drosophila)
9.8 × 10^−7^	1.8	Klhl12	kelch-like 12 (Drosophila)
1.8 × 10^−8^	1.8	Dusp5	dual specificity phosphatase 5
6.7 × 10^−3^	1.8	Sema3d	sema domain, immunoglobulin domain (Ig), short basic domain, secreted, (semaphorin) 3D
9.6 × 10^−3^	1.7	1700042O10Rik	RIKEN cDNA 1700042O10 gene
1.4 × 10^−6^	1.7	Cmah	cytidine monophospho-N-acetylneuraminic acid hydroxylase
1.4 × 10^−5^	1.7	Pilrb1	paired immunoglobin-like type 2 receptor beta 1
1.2 × 10^−4^	1.7	Vangl2	vang-like 2 (van gogh, Drosophila)
2.1 × 10^−7^	1.7	Ikbke	inhibitor of kappaB kinase epsilon
4.9 × 10^−8^	1.7	Fam20c	family with sequence similarity 20, member C
8.9 × 10^−8^	1.7	Tagap	T cell activation Rho GTPase activating protein
2.5 × 10^−7^	1.6	Mafb	v-maf musculoaponeurotic fibrosarcoma oncogene family, protein B (avian)
3.9 × 10^−4^	1.6	Vdr	vitamin D receptor
5.4 × 10^−7^	1.6	Gda	guanine deaminase
5.3 × 10^−5^	1.6	Fbxo32	F-box protein 32
1.2 × 10^−5^	1.6	Gm16010	predicted gene 16010
2.0 × 10^−7^	1.6	Bcl3	B cell leukemia/lymphoma 3
1.9 × 10^−7^	1.6	Hcfc2	host cell factor C2
1.4 × 10^−6^	1.6	Cd97	CD97 antigen
1.6 × 10^−7^	1.6	Lfng	LFNG O-fucosylpeptide 3-beta-N-acetylglucosaminyltransferase
4.2 × 10^−6^	1.5	Aifm2	apoptosis-inducing factor, mitochondrion-associated 2
1.8 × 10^−6^	1.5	Spsb4	splA/ryanodine receptor domain and SOCS box containing 4
**Downregulated Transcripts**			
**Corr. *P* val.**	**FC**	**Gene Symbol**	**Gene Title**
2.3 × 10^−3^	−4.1	Klf5	Kruppel-like factor 5
1.3 × 10^−3^	−3.9	Zfp831	zinc finger protein 831
6.8 × 10^−3^	−3.6	St8sia6	ST8 alpha-N-acetyl-neuraminide alpha-2,8-sialyltransferase 6
3.6 × 10^−4^	−3.2	Gm1564	predicted gene 1564
9.3 × 10^−5^	−3.1	Efna1	ephrin A1
1.2 × 10^−3^	−3.0	Plat	plasminogen activator, tissue
1.0 × 10^−2^	−2.9	Ucp3	uncoupling protein 3 (mitochondrial, proton carrier)
2.6 × 10^−3^	−2.7	Havcr1	hepatitis A virus cellular receptor 1
5.4 × 10^−3^	−2.7	Heyl	hairy/enhancer-of-split related with YRPW motif-like
1.7 × 10^−3^	−2.7	Akr1b7	aldo-keto reductase family 1, member B7
5.6 × 10^−3^	−2.5	Mir425	microRNA 425
2.5 × 10^−4^	−2.4	Ighv6-3	immunoglobulin heavy variable 6-3
2.5 × 10^−3^	−2.2	Cyp4f41-ps	cytochrome P450, family 4, subfamily f, polypeptide 41 pseudogene
4.7 × 10^−5^	−2.2	Emr4	EGF-like module containing, mucin-like, hormone receptor-like sequence 4
9.4 × 10^−5^	−2.1	Styk1	serine/threonine/tyrosine kinase 1
2.0 × 10^−4^	−2.1	Gpr182	G protein-coupled receptor 182
1.2 × 10^−5^	−2.1	U6	U6 spliceosomal RNA
1.8 × 10^−4^	−2.0	Ch25h	cholesterol 25-hydroxylase
8.2 × 10^−9^	−1.9	Rnd3	Rho family GTPase 3
1.7 × 10^−2^	−1.8	Gm6776	predicted pseudogene 6776
1.1 × 10^−2^	−1.8	Pxdc1	PX domain containing 1
1.2 × 10^−4^	−1.8	Socs3	suppressor of cytokine signaling 3
1.7 × 10^−6^	−1.8	Tnf	tumor necrosis factor
1.8 × 10^−10^	−1.8	Cited2	Cbp/p300-interacting transactivator, with Glu/Asp-rich carboxy-terminal domain, 2
3.1 × 10^−6^	−1.6	Spata13	spermatogenesis associated 13
1.8 × 10^−8^	−1.6	Rasgef1b	RasGEF domain family, member 1B
7.3 × 10^−6^	−1.6	Gpr85	G protein-coupled receptor 85
1.5 × 10^−4^	−1.6	Rgs7bp	regulator of G-protein signaling 7 binding protein
1.3 × 10^−7^	−1.5	Dusp1	dual specificity phosphatase 1
1.2 × 10^−3^	−1.5	Tnfaip3	tumor necrosis factor, alpha-induced protein 3
1.9 × 10^−3^	−1.5	Gm16541	predicted gene 16541
4.7 × 10^−6^	−1.5	Tmem178	transmembrane protein 178

**Table 3:** List of the upregulated M2 macrophage-associated genes differentially expressed between 48 h DMSO- and 48 h DHR-treated BMDMs (based on at least 1.5-fold change and corrected *p* value < 0.05). M2 macrophage-associated list containing 73 genes was generated based on the https://www.bosterbio.com/tissue-markers-cell-markers/macrophage-markers (accessed on 9 August 2022) website and extended with genes from literature search and cross-referenced with the 357 genes, which were upregulated following 48 h DHR-treatment.

Corr. *p* Val.	FC	Gene Symbol	Gene Title
8.93 × 10^−8^	249.6	Bmp2	bone morphogenetic protein 2
1.83 × 10^−4^	14.8	Cyp26b1	cytochrome P450, family 26, subfamily b, polypeptide 1
9.13 × 10^−7^	4.4	Vegfa	vascular endothelial growth factor A
3.76 × 10^−4^	4.1	Aldh1a2	aldehyde dehydrogenase family 1, subfamily A2
4.38 × 10^−3^	3.6	Marco	macrophage receptor with collagenous structure
4.85 × 10^−7^	2.8	Clec7a	C-type lectin domain family 7, member a
2.01 × 10^−4^	1.9	Irf4	interferon regulatory factor 4
5.56 × 10^−5^	1.7	Siglec1	sialic acid binding Ig-like lectin 1, sialoadhesin
6.24 × 10^−8^	1.6	Tgm2	transglutaminase 2, C polypeptide
1.43 × 10^−4^	1.5	Cd36	CD36 antigen

## Data Availability

The raw and log2 normalized RNA sequencing data can be found in the Supplementary Materials.

## Supplementary Materials

The following supporting information can be downloaded at: https://www.mdpi.com/article/10.3390/cells11182928/s1, Table S1: List of 357 transcripts which were significantly upregulated in BMDMs exposed to 1 μM DHR during the last 2 days of their differentiation compared to DMSO treatment (based on at least 1.5-fold change and corrected *p* value < 0.05); Table S2: List of 424 transcripts which were significantly downregulated in BMDMs exposed to 1 μM DHR during the last 2 days of their differentiation compared to DMSO treatment (based on at least 1.5-fold change and corrected *p* value < 0.05); Table S3: List of the 30 most strongly enriched Gene Ontology Biological process terms among the 357 transcripts that were upregulated in the 48h DHR-treated BMDMs compared to the DMSO-treated ones; Table S4: List of 102 differently expressed transcripts between 2 h DMSO- or 2 h DHR (1 μM)-treated 4 day-differentiated monocytes (based on at least 1.5-fold change and corrected *p* value < 0.05).
