# Supplementary material for: Retinoids Promote Mouse Bone Marrow-Derived Macrophage Differentiation and Efferocytosis via Upregulating Bone Morphogenetic Protein-2 and Smad3
Source: Cells. 2022 Sep 19;11(18):2928. doi: 10.3390/cells11182928 (PMC9497139; doi:10.3390/cells11182928)
Supplement: Supplementary file 1 [file cells-11-02928-s001.zip › cells-1885345-supplementary/Supplementary tables.pdf]

Supplementary Table S1. List of 357 transcripts which were significantly upregulated in BMDMs exposed to 1 μM DHR during the last 2 days of their differentiation compared to DMSO treatment (based on at least 1.5-fold change and corrected p value<0.05).

Corr. p val.	FC	Gene symbol	Gene title
8.9E-08	249.6	Bmp2	bone morphogenetic protein 2
1.2E-06	212.5	Camkk1	calcium/calmodulin-dependent protein kinase kinase 1, alpha
1.2E-06	193.0	Mmp9	matrix metallopeptidase 9
7.1E-06	168.1	Rarb	retinoic acid receptor, beta
2.1E-05	70.6	B230378P21Rik	RIKEN cDNA B230378P21 gene
1.6E-07	51.5	Cd38	CD38 antigen
3.1E-05	45.9	Slc12a5	solute carrier family 12, member 5
7.4E-05	37.5	Chst15	carbohydrate (N-acetylgalactosamine 4-sulfate 6-O) sulfotransferase 15
2.0E-07	35.4	Gm13431	predicted gene 13431
4.7E-06	28.7	Col14a1	collagen, type XIV, alpha 1
2.9E-06	28.5	Cxcl14	chemokine (C-X-C motif) ligand 14
4.9E-08	26.8	Pram1	PML-RAR alpha-regulated adaptor molecule 1
4.3E-05	26.2	Kcnip3	Kv channel interacting protein 3, calsenilin
1.8E-08	23.9	Hic1	hypermethylated in cancer 1
5.1E-04	22.8	Tspan18	tetraspanin 18
7.8E-05	22.7	Fgf1	fibroblast growth factor 1
3.2E-06	21.0	Art2a-ps	ADP-ribosyltransferase 2a, pseudogene
8.0E-08	18.9	Csn3	casein kappa
7.5E-05	15.9	Pde1c	phosphodiesterase 1C
3.7E-07	15.6	Prtn3	proteinase 3
1.8E-04	14.8	Cyp26b1	cytochrome P450, family 26, subfamily b, polypeptide 1
1.4E-07	14.0	Ednrb	endothelin receptor type B
1.3E-10	11.2	Ptgs1	prostaglandin-endoperoxide synthase 1
1.6E-03	10.8	Fam20a	family with sequence similarity 20, member A
5.2E-08	10.7	Bcl2a1b	B cell leukemia/lymphoma 2 related protein A1b
1.7E-04	10.4	Plxna4	plexin A4
8.7E-07	9.9	Col4a5	collagen, type IV, alpha 5
7.4E-05	9.5	BC035044	cDNA sequence BC035044
1.3E-06	9.3	BC005764	cDNA sequence BC005764
1.1E-04	9.0	Mdfi	MyoD family inhibitor
5.1E-04	8.9	Gpr35	G protein-coupled receptor 35
1.3E-07	8.9	Ccl6	chemokine (C-C motif) ligand 6
1.4E-06	8.4	Pkd1l2	polycystic kidney disease 1 like 2

2.2E-03	8.4	P2rx1	purinergic receptor P2X, ligand-gated ion channel, 1
4.5E-09	8.2	Acp5	acid phosphatase 5, tartrate resistant
4.3E-06	7.9	Asb4	ankyrin repeat and SOCS box-containing 4
2.3E-05	7.7	Hs3st3a1	heparan sulfate (glucosamine) 3-O-sulfotransferase 3A1
8.8E-09	7.6	Card11	caspase recruitment domain family, member 11
1.2E-07	7.6	Rbpms	RNA binding protein gene with multiple splicing
9.2E-04	7.4	Il1a	interleukin 1 alpha
1.0E-07	7.3	Ifitm6	interferon induced transmembrane protein 6
2.0E-05	7.3	Hs3st3b1	heparan sulfate (glucosamine) 3-O-sulfotransferase 3B1
9.9E-03	7.0	Col4a6	collagen, type IV, alpha 6
1.6E-07	6.9	Fcna	ficolin A
5.0E-03	6.9	Gm10567	predicted gene 10567
1.1E-04	6.7	Sell	selectin, lymphocyte
4.6E-06	6.6	Ryr1	ryanodine receptor 1, skeletal muscle
6.9E-07	6.5	Il21r	interleukin 21 receptor
2.4E-09	6.4	Ltbp3	latent transforming growth factor beta binding protein 3
2.6E-05	6.1	Cfhr2	complement factor H-related 2
1.8E-03	6.0	Gm4788	predicted gene 4788
3.7E-05	6.0	Prss50	protease, serine, 50
3.9E-09	5.9	Gm20507	predicted gene 20507
3.6E-07	5.8	Mfng	MFNG O-fucosylpeptide 3-beta-N-acetylglucosaminyltransferase
2.0E-03	5.8	2010320O07Rik	RIKEN cDNA 2010320O07 gene
1.7E-02	5.7	Foxq1	forkhead box Q1
1.5E-04	5.6	Clec1a	C-type lectin domain family 1, member a
2.0E-02	5.1	Ddit4l	DNA-damage-inducible transcript 4-like
2.0E-06	5.1	Socs2	suppressor of cytokine signaling 2
1.6E-03	5.0	BC021767	cDNA sequence BC021767
3.2E-07	4.9	Pde2a	phosphodiesterase 2A, cGMP-stimulated
7.1E-06	4.9	Gm16332	predicted gene 16332
7.6E-06	4.8	Enpp4	ectonucleotide pyrophosphatase/phosphodiesterase 4
1.6E-05	4.7	Treml2	triggering receptor expressed on myeloid cells-like 2
4.0E-08	4.7	Bcl2a1d	B cell leukemia/lymphoma 2 related protein A1d
1.8E-06	4.5	Bcl2a1a	B cell leukemia/lymphoma 2 related protein A1a
2.1E-07	4.5	Hivep2	human immunodeficiency virus type I enhancer binding protein 2
1.5E-06	4.5	Fam102a	family with sequence similarity 102, member A
4.8E-03	4.5	Cgn	cingulin
6.3E-07	4.5	Cfh	complement component factor h

9.1E-07	4.4	Vegfa	vascular endothelial growth factor A
6.7E-03	4.3	B630019A10Rik	RIKEN cDNA B630019A10 gene
1.1E-06	4.3	Cd24a	CD24a antigen
4.4E-03	4.2	Art2b	ADP-ribosyltransferase 2b
1.7E-03	4.2	Gm16268	predicted gene 16268
3.0E-04	4.2	Fabp7	fatty acid binding protein 7, brain
1.0E-07	4.1	Smad3	SMAD family member 3
3.8E-04	4.1	Aldh1a2	aldehyde dehydrogenase family 1, subfamily A2
3.4E-04	4.1	Grtp1	GH regulated TBC protein 1
1.4E-04	4.0	A430108G06Rik	RIKEN cDNA A430108G06 gene
2.6E-09	3.9	2510009E07Rik	RIKEN cDNA 2510009E07 gene
2.1E-07	3.9	Ikbke	inhibitor of kappaB kinase epsilon
5.2E-03	3.9	Wnk2	WNK lysine deficient protein kinase 2
2.3E-06	3.9	Epas1	endothelial PAS domain protein 1
5.7E-04	3.9	Flywch2	FLYWCH family member 2
2.5E-06	3.8	Gm22	predicted gene 22
8.8E-09	3.8	Dtx4	deltex 4 homolog (Drosophila)
8.6E-03	3.8	Enpp5	ectonucleotide pyrophosphatase/phosphodiesterase 5
5.4E-05	3.7	Nes	nestin
5.4E-07	3.7	Gda	guanine deaminase
4.4E-03	3.6	Marco	macrophage receptor with collagenous structure
1.4E-05	3.6	Pilrb1	paired immunoglobin-like type 2 receptor beta 1
1.3E-07	3.6	Rab20	RAB20, member RAS oncogene family
1.2E-06	3.6	Cp	ceruloplasmin
2.4E-03	3.5	Sytl3	synaptotagmin-like 3
1.5E-02	3.5	Gm11724	predicted gene 11724
8.8E-07	3.5	Apoc2	apolipoprotein C-II
9.4E-04	3.4	Onecut3	one cut domain, family member 3
1.7E-03	3.4	Gm15635	predicted gene 15635
1.1E-02	3.4	Gpr31b	G protein-coupled receptor 31, D17Leh66b region
1.1E-03	3.3	Phf11	PHD finger protein 11
8.6E-05	3.3	Hbegf	heparin-binding EGF-like growth factor
3.9E-03	3.3	Ascl2	achaete-scute complex homolog 2 (Drosophila)
4.9E-09	3.3	Anxa3	annexin A3
8.4E-07	3.2	Pilrb2	paired immunoglobin-like type 2 receptor beta 2
1.0E-03	3.2	Gm13337	predicted gene 13337
1.1E-03	3.2	Stab2	stabilin 2
9.8E-07	3.2	Klhl12	kelch-like 12 (Drosophila)
1.2E-05	3.2	Gm16010	predicted gene 16010
4.9E-06	3.1	Stat4	signal transducer and activator of transcription 4
2.9E-03	3.1	Ccdc48	coiled-coil domain containing 48
1.4E-02	3.1	Smtnl2	smoothelin-like 2

2.3E-05	3.1	Gm11464	predicted gene 11464
8.9E-08	3.1	Tagap	T cell activation Rho GTPase activating protein
1.1E-02	3.0	Gsta4	glutathione S-transferase, alpha 4
6.7E-03	3.0	Asb2	ankyrin repeat and SOCS box-containing 2
4.9E-08	3.0	Fam20c	family with sequence similarity 20, member C
3.0E-03	3.0	1700024P16Rik	RIKEN cDNA 1700024P16 gene
4.1E-04	3.0	Htra1	HtrA serine peptidase 1
3.0E-03	3.0	Gm15395	predicted gene 15395
1.4E-06	3.0	Cd97	CD97 antigen
4.5E-03	2.9	Ak3l2-ps	adenylate kinase 3-like 2, pseudogene
6.5E-08	2.9	Osgin1	oxidative stress induced growth inhibitor 1
3.3E-05	2.9	Npm2	nucleophosmin/nucleoplasmin 2
2.5E-06	2.8	Palm	paralemmin
5.2E-03	2.8	Egln3	EGL nine homolog 3 (C. elegans)
1.9E-04	2.8	5031425F14Rik	RIKEN cDNA 5031425F14 gene
8.3E-04	2.8	Tubb3	tubulin, beta 3 class III
4.9E-07	2.8	Clec7a	C-type lectin domain family 7, member a
2.7E-04	2.8	Prrg4	proline rich Gla (G-carboxyglutamic acid) 4 (transmembrane)
1.2E-03	2.8	Akap2	A kinase (PRKA) anchor protein 2
6.7E-03	2.8	A530084C06Rik	RIKEN cDNA A530084C06 gene
4.0E-04	2.7	Gm20459	predicted gene 20459
3.8E-03	2.7	Aldh1l2	aldehyde dehydrogenase 1 family, member L2
6.1E-06	2.7	Ccnd2	cyclin D2
5.2E-06	2.7	Tmc8	transmembrane channel-like gene family 8
5.5E-03	2.7	Gm6904	predicted gene 6904
1.4E-07	2.7	Clec2i	C-type lectin domain family 2, member i
2.2E-07	2.7	Baiap2	brain-specific angiogenesis inhibitor 1-associated protein 2
1.5E-03	2.7	Osbp2	oxysterol binding protein 2
1.6E-04	2.7	Vsig8	V-set and immunoglobulin domain containing 8
3.5E-07	2.7	Sdc1	syndecan 1
1.3E-05	2.7	AA467197	expressed sequence AA467197
1.3E-02	2.6	Gpr124	G protein-coupled receptor 124
1.0E-07	2.6	Cd53	CD53 antigen
1.2E-07	2.6	Gsta3	glutathione S-transferase, alpha 3
3.7E-07	2.6	Ltc4s	leukotriene C4 synthase
3.1E-07	2.6	B430306N03Rik	RIKEN cDNA B430306N03 gene
3.9E-04	2.6	H2afy2	H2A histone family, member Y2
1.2E-03	2.6	Gm4902	predicted gene 4902
1.3E-06	2.6	Rap1gap2	RAP1 GTPase activating protein 2
1.6E-03	2.6	Plat	plasminogen activator, tissue

1.6E-04	2.6	Gas2	growth arrest specific 2
8.1E-05	2.6	Cmtm8	CKLF-like MARVEL transmembrane domain containing 8
7.1E-04	2.6	Syt8	synaptotagmin VIII
1.8E-02	2.6	Rpl31-ps17	ribosomal protein L31, pseudogene 17
1.9E-03	2.6	Corin	corin
1.6E-06	2.5	Gm4980	predicted gene 4980
3.2E-06	2.5	Slco2b1	solute carrier organic anion transporter family, member 2b1
1.2E-08	2.5	1810011H11Rik	RIKEN cDNA 1810011H11 gene
1.6E-05	2.5	Ddah2	dimethylarginine dimethylaminohydrolase 2
1.8E-02	2.5	4833418N02Rik	RIKEN cDNA 4833418N02 gene
7.9E-07	2.5	Slc40a1	solute carrier family 40 (iron-regulated transporter), member 1
5.2E-06	2.5	Sox4	SRY-box containing gene 4
1.5E-02	2.5	Ak4	adenylate kinase 4
1.5E-04	2.5	Palld	palladin, cytoskeletal associated protein
4.9E-08	2.4	Parvg	parvin, gamma
5.2E-06	2.4	Myh10	myosin, heavy polypeptide 10, non-muscle
4.4E-06	2.4	Dnmt3l	DNA (cytosine-5-)-methyltransferase 3-like
1.5E-05	2.4	Gm20658	predicted gene 20658
6.2E-05	2.4	Rasgrp2	RAS, guanyl releasing protein 2
4.2E-06	2.4	Aifm2	apoptosis-inducing factor, mitochondrion-associated 2
4.6E-05	2.4	Bspry	B-box and SPRY domain containing
1.4E-02	2.4	Cand2	cullin-associated and neddylation-dissociated 2 (putative)
7.2E-07	2.4	Mt2	metallothionein 2
4.3E-07	2.4	Fcgr4	Fc receptor, IgG, low affinity IV
1.1E-09	2.4	Mcart1	mitochondrial carrier triple repeat 1
2.2E-03	2.4	Rap1gap	Rap1 GTPase-activating protein
2.7E-06	2.4	Rai14	retinoic acid induced 14
2.2E-04	2.4	Mdm1	transformed mouse 3T3 cell double minute 1
7.7E-04	2.4	Gm9252	predicted gene 9252
5.5E-04	2.4	Gm6377	predicted gene 6377
2.5E-06	2.3	Ndst1	N-deacetylase/N-sulfotransferase (heparan glucosaminyl) 1
1.3E-08	2.3	Tbxas1	thromboxane A synthase 1, platelet
1.2E-04	2.3	Vangl2	vang-like 2 (van gogh, Drosophila)
1.2E-05	2.3	St3gal5	ST3 beta-galactoside alpha-2,3-sialyltransferase 5
1.8E-06	2.3	Spsb4	splA/ryanodine receptor domain and SOCS box containing 4

1.6E-07	2.3	Ubxn8	UBX domain protein 8
2.6E-04	2.3	Soat2	sterol O-acyltransferase 2
5.4E-06	2.3	Tpt1-ps5	tumor protein, translationally-controlled, pseudogene 5
1.3E-02	2.3	Smo	smoothened homolog (Drosophila)
3.8E-08	2.3	Rhoc	ras homolog gene family, member C
1.6E-04	2.3	E430024P14Rik	RIKEN cDNA E430024P14 gene
8.4E-06	2.3	Gpc1	glypican 1
2.1E-06	2.3	Dchs1	dachsous 1 (Drosophila)
2.2E-07	2.3	Traf3ip3	TRAF3 interacting protein 3
1.0E-07	2.3	Hdac9	histone deacetylase 9
7.0E-03	2.3	Mettl21c	methyltransferase like 21C
1.1E-02	2.3	Gm8724	predicted pseudogene 8724
2.2E-05	2.3	Ifitm3	interferon induced transmembrane protein 3
5.8E-05	2.3	D330020A13RIK	Putative uncharacterized protein
7.0E-09	2.3	Gsr	glutathione reductase
4.0E-06	2.3	Ccrl2	chemokine (C-C motif) receptor-like 2
4.5E-08	2.3	Fam117a	family with sequence similarity 117, member A
3.2E-03	2.2	Cxcr5	chemokine (C-X-C motif) receptor 5
3.5E-07	2.2	Map4k1	mitogen-activated protein kinase kinase kinase kinase 1
7.7E-03	2.2	Aldh5a1	aldhehyde dehydrogenase family 5, subfamily A1
2.5E-06	2.2	Prom1	prominin 1
1.0E-04	2.2	Gm11521	predicted gene 11521
9.4E-08	2.2	Nrip1	nuclear receptor interacting protein 1
1.9E-07	2.2	Runx3	runt related transcription factor 3
6.9E-08	2.2	Neurl3	neuralized homolog 3 homolog (Drosophila)
5.1E-06	2.1	Rhou	ras homolog gene family, member U
1.0E-03	2.1	AF529169	cDNA sequence AF529169
4.1E-05	2.1	Samsn1	SAM domain, SH3 domain and nuclear localization signals, 1
1.8E-02	2.1	Gm15701	predicted gene 15701
3.2E-06	2.1	Pitpnc1	phosphatidylinositol transfer protein, cytoplasmic 1
2.0E-08	2.1	Phf15	PHD finger protein 15
4.4E-04	2.1	H1fx	H1 histone family, member X
3.8E-04	2.1	Cd1d1	CD1d1 antigen
4.1E-03	2.1	March9	membrane-associated ring finger (C3HC4) 9
6.5E-08	2.1	Kpna4	karyopherin (importin) alpha 4
2.8E-03	2.1	Gm14027	predicted gene 14027
5.8E-03	2.0	C330011M18Rik	RIKEN cDNA C330011M18 gene
1.4E-07	2.0	Prdx5	peroxiredoxin 5
2.3E-03	2.0	0610010O12Rik	RIKEN cDNA 0610010O12 gene

1.7E-04	2.0	Bank1	B cell scaffold protein with ankyrin repeats 1
2.1E-03	2.0	Galnt3	UDP-N-acetyl-alpha-D-galactosamine:polypeptide N-acetylgalactosaminyltransferase 3
1.8E-05	2.0	Gm7901	predicted gene 7901
5.3E-05	2.0	Fbxo32	F-box protein 32
1.5E-06	2.0	Padi4	peptidyl arginine deiminase, type IV
7.0E-03	2.0	Dmwd	dystrophia myotonica-containing WD repeat motif
1.0E-07	2.0	Gatm	glycine amidinotransferase (L-arginine:glycine amidinotransferase)
8.4E-05	2.0	Gm17114	predicted gene 17114
1.7E-03	2.0	1700062I23Rik	RIKEN cDNA 1700062I23 gene
4.9E-04	2.0	Thbs1	thrombospondin 1
2.7E-03	2.0	Ccdc148	coiled-coil domain containing 148
6.7E-04	2.0	Rec8	REC8 homolog (yeast)
1.9E-03	2.0	Olfr1417	olfactory receptor 1417
4.5E-04	2.0	Pilra	paired immunoglobin-like type 2 receptor alpha
4.1E-06	2.0	Insig1	insulin induced gene 1
8.7E-07	2.0	Itpr3	inositol 1,4,5-triphosphate receptor 3
1.1E-03	1.9	Prr5	proline rich 5 (renal)
5.3E-08	1.9	Ninj1	ninjurin 1
5.6E-08	1.9	Tpt1	tumor protein, translationally-controlled 1
2.7E-04	1.9	Kif19a	kinesin family member 19A
1.6E-08	1.9	Tpt1-ps3	tumor protein, translationally-controlled, pseudogene 3
2.0E-04	1.9	Irf4	interferon regulatory factor 4
2.0E-05	1.9	Arrdc3	arrestin domain containing 3
3.3E-04	1.9	Ctse	cathepsin E
1.4E-04	1.9	Clic5	chloride intracellular channel 5
1.9E-07	1.9	Tpt1-ps6	tumor protein, translationally-controlled, pseudogene 6
1.5E-08	1.9	Kif3a	kinesin family member 3A
3.2E-06	1.9	Phgdh	3-phosphoglycerate dehydrogenase
6.8E-07	1.9	Gadd45a	growth arrest and DNA-damage-inducible 45 alpha
8.5E-04	1.9	Acy1	aminoacylase 1
8.2E-04	1.9	Per2	period homolog 2 (Drosophila)
4.4E-05	1.9	Fam65b	family with sequence similarity 65, member B
1.4E-06	1.9	Tagap1	T cell activation GTPase activating protein 1
1.3E-09	1.9	Slc7a5	solute carrier family 7 (cationic amino acid transporter, y+ system), member 5
7.1E-07	1.9	Slc30a9	solute carrier family 30 (zinc transporter), member 9
5.7E-08	1.9	Chac1	ChaC, cation transport regulator 1
8.1E-03	1.8	Gm11201	predicted gene 11201

3.8E-09	1.8	Tubb6	tubulin, beta 6 class V
3.3E-03	1.8	Gm5540	predicted pseudogene 5540
4.8E-05	1.8	Prkcb	protein kinase C, beta
1.1E-09	1.8	St6gal1	beta galactoside alpha 2,6 sialyltransferase 1
1.8E-06	1.8	Cgnl1	cingulin-like 1
9.0E-07	1.8	Scn1b	sodium channel, voltage-gated, type I, beta
9.5E-04	1.8	Eps8	epidermal growth factor receptor pathway substrate 8
2.2E-08	1.8	Man1a	mannosidase 1, alpha
7.0E-07	1.8	Mov10	Moloney leukemia virus 10
1.5E-05	1.8	Dip2c	DIP2 disco-interacting protein 2 homolog C (Drosophila)
6.9E-06	1.8	Acsf2	acyl-CoA synthetase family member 2
4.1E-07	1.8	Rnaset2a	ribonuclease T2A
2.7E-06	1.8	Fam125b	family with sequence similarity 125, member B
8.3E-05	1.8	Gadd45g	growth arrest and DNA-damage-inducible 45 gamma
8.8E-09	1.8	Rasgrp3	RAS, guanyl releasing protein 3
6.9E-06	1.8	Jdp2	Jun dimerization protein 2
1.4E-06	1.8	Cmah	cytidine monophospho-N-acetylneuraminic acid hydroxylase
1.5E-08	1.8	Fam134b	family with sequence similarity 134, member B
8.7E-06	1.8	Siglec5	sialic acid binding Ig-like lectin 5
1.1E-08	1.7	P2rx7	purinergic receptor P2X, ligand-gated ion channel, 7
1.9E-07	1.7	Hcfc2	host cell factor C2
9.8E-06	1.7	Agpat9	1-acylglycerol-3-phosphate O-acyltransferase 9
1.6E-07	1.7	Lfng	LFNG O-fucosylpeptide 3-beta-N-acetylglucosaminyltransferase
2.5E-06	1.7	Slc1a4	solute carrier family 1 (glutamate/neutral amino acid transporter), member 4
7.9E-05	1.7	Susd1	sushi domain containing 1
6.2E-06	1.7	Zfp238	zinc finger protein 238
8.7E-07	1.7	Itch	itchy, E3 ubiquitin protein ligase
3.1E-07	1.7	Rnaset2b	ribonuclease T2B
7.4E-03	1.7	Arhgap32	Rho GTPase activating protein 32
2.0E-04	1.7	Klhl38	kelch-like 38 (Drosophila)
1.4E-05	1.7	Hrh1	histamine receptor H1
3.1E-03	1.7	4930455G09Rik	RIKEN cDNA 4930455G09 gene
1.8E-05	1.7	Ets2	E26 avian leukemia oncogene 2, 3' domain
1.4E-03	1.7	Tnnt1	troponin T1, skeletal, slow
1.4E-04	1.7	Mex3b	mex3 homolog B (C. elegans)
9.4E-06	1.7	Trf	transferrin

6.1E-03	1.7	Gm14719	predicted gene 14719
1.4E-05	1.7	Ppdpf	pancreatic progenitor cell differentiation and proliferation factor homolog (zebrafish)RIKEN cDNA 2700038C09 gene
5.6E-05	1.7	Siglec1	sialic acid binding Ig-like lectin 1, sialoadhesin
5.3E-07	1.7	Gm20425	predicted gene 20425
3.6E-07	1.7	Plk2	polo-like kinase 2
1.8E-05	1.6	Ccr1	chemokine (C-C motif) receptor 1
1.5E-06	1.6	Atf5	activating transcription factor 5
2.6E-03	1.6	4933427G23Rik	RIKEN cDNA 4933427G23 gene
1.3E-05	1.6	Dhdh	dihydrodiol dehydrogenase (dimeric)
2.2E-08	1.6	Tnfaip8	tumor necrosis factor, alpha-induced protein 8
1.2E-05	1.6	Sap30	sin3 associated polypeptide
5.1E-07	1.6	Nup210	nucleoporin 210
2.1E-06	1.6	Rnf141	ring finger protein 141
1.2E-06	1.6	Atxn2	ataxin 2
4.6E-07	1.6	4931406C07Rik	RIKEN cDNA 4931406C07 gene
7.8E-03	1.6	Wnt6	wingless-related MMTV integration site 6
6.7E-08	1.6	Asns	asparagine synthetase
3.3E-05	1.6	Hoxb4	homeobox B4
2.3E-07	1.6	Il16	interleukin 16
2.0E-04	1.6	Grap	GRB2-related adaptor protein
7.1E-03	1.6	Spp1	secreted phosphoprotein 1
2.8E-04	1.6	Nhsl2	NHS-like 2
7.8E-04	1.6	Fzd1	frizzled homolog 1 (Drosophila)
6.2E-08	1.6	Tgm2	transglutaminase 2, C polypeptide
3.7E-05	1.6	Slc25a23	solute carrier family 25 (mitochondrial carrier; phosphate carrier), member 23
1.6E-05	1.6	Ly86	lymphocyte antigen 86
6.9E-04	1.6	Morn4	MORN repeat containing 4
1.2E-05	1.6	Enc1	ectodermal-neural cortex 1
2.6E-06	1.6	Zfp503	zinc finger protein 503
2.9E-06	1.6	Zdhhc14	zinc finger, DHHC domain containing 14
4.4E-04	1.6	Zfp438	zinc finger protein 438
5.6E-07	1.6	Myo7a	myosin VIIA
6.2E-06	1.6	4930481A15Rik	RIKEN cDNA 4930481A15 gene
1.0E-07	1.6	Slc2a6	solute carrier family 2 (facilitated glucose transporter), member 6
1.2E-03	1.6	Plcxd2	phosphatidylinositol-specific phospholipase C, X domain containing 2
8.2E-07	1.6	Kitl	kit ligand
5.2E-03	1.6	Axl	AXL receptor tyrosine kinase

2.5E-07	1.6	Dusp6	dual specificity phosphatase 6
1.4E-03	1.6	Tmem42	transmembrane protein 42
9.5E-08	1.5	Ccl2	chemokine (C-C motif) ligand 2
3.4E-04	1.5	Bex1	brain expressed gene 1
1.5E-06	1.5	Pde7a	phosphodiesterase 7A
1.5E-07	1.5	Hs6st1	heparan sulfate 6-O-sulfotransferase 1
1.1E-06	1.5	Tgs1	trimethylguanosine synthase homolog (S. cerevisiae)
1.9E-03	1.5	Gm16363	predicted gene 16363
1.0E-06	1.5	Cdc42ep2	CDC42 effector protein (Rho GTPase binding) 2
6.7E-07	1.5	A530064D06Rik	RIKEN cDNA A530064D06 gene
1.4E-04	1.5	Cd36	CD36 antigen
2.7E-03	1.5	Mthfd2l	methylenetetrahydrofolate dehydrogenase (NADP+ dependent) 2-like
7.4E-05	1.5	Arap2	ArfGAP with RhoGAP domain, ankyrin repeat and PH domain 2
1.7E-06	1.5	Ccdc88b	coiled-coil domain containing 88B
5.8E-09	1.5	Cnrip1	cannabinoid receptor interacting protein 1
5.7E-03	1.5	Bach2	BTB and CNC homology 2
1.0E-04	1.5	Tecpr2	tectonin beta-propeller repeat containing 2
2.0E-08	1.5	Ehd1	EH-domain containing 1
4.0E-04	1.5	Tmem141	transmembrane protein 141
8.5E-03	1.5	Adat2	adenosine deaminase, tRNA-specific 2
1.5E-07	1.5	Trib3	tribbles homolog 3 (Drosophila)

Supplementary Table S2. List of 424 transcripts which were significantly downregulated in BMDMs exposed to 1 μM DHR during the last 2 days of their differentiation compared to DMSO treatment (based on at least 1.5-fold change and corrected p value<0.05).

Corr. p val.	FC	Gene symbol	Gene title
1.2E-08	-23.4	Olfml3	olfactomedin-like 3
2.9E-04	-16.5	Nptx1	neuronal pentraxin 1
1.8E-07	-14.8	Cd276	CD276 antigen
5.9E-04	-9.8	Slc6a4	solute carrier family 6 (neurotransmitter transporter, serotonin), member 4
7.7E-07	-9.2	Gp5	glycoprotein 5 (platelet)
1.5E-04	-9.0	Gm10134	predicted gene 10134
2.5E-03	-8.0	Mtap2	microtubule-associated protein 2
1.9E-06	-8.0	Gata1	GATA binding protein 1
5.7E-03	-8.0	Kcnj16	potassium inwardly-rectifying channel, subfamily J, member 16
5.0E-03	-7.6	Pcsk1	proprotein convertase subtilisin/kexin type 1

1.8E-05	-7.4	Gucy1b3	guanylate cyclase 1, soluble, beta 3
6.5E-06	-7.2	Ehd3	EH-domain containing 3
4.8E-06	-7.1	Clu	clusterin
5.9E-04	-6.9	Cd207	CD207 antigen
3.3E-05	-6.5	F2rl2	coagulation factor II (thrombin) receptor-like 2
4.5E-08	-6.2	Myct1	myc target 1
1.1E-04	-6.2	Atp2b4	ATPase, Ca++ transporting, plasma membrane 4
1.3E-05	-6.1	Gucy1a3	guanylate cyclase 1, soluble, alpha 3
4.5E-05	-5.8	Kcnj5	potassium inwardly-rectifying channel, subfamily J, member 5
5.0E-05	-5.8	Cd226	CD226 antigen
4.0E-06	-5.5	Pde3a	phosphodiesterase 3A, cGMP inhibited
1.5E-02	-5.5	Tmem246	transmembrane protein 246
5.2E-07	-5.4	Gbgt1	globoside alpha-1,3-N-acetylgalactosaminyltransferase 1
9.1E-05	-5.3	Cd300e	CD300e antigen
8.8E-06	-5.3	Gp6	glycoprotein 6 (platelet)
5.7E-07	-5.3	Snca	synuclein, alpha
4.5E-07	-4.8	Ppbp	pro-platelet basic protein
9.6E-07	-4.7	Sdpr	serum deprivation response
7.2E-07	-4.7	Nrgn	neurogranin
1.6E-05	-4.6	Faim3	Fas apoptotic inhibitory molecule 3
6.3E-04	-4.6	Gm16033	predicted gene 16033
4.2E-04	-4.6	Gm16282	predicted gene 16282
2.9E-06	-4.6	Cxcl5	chemokine (C-X-C motif) ligand 5
9.3E-03	-4.5	Rep15	RAB15 effector protein
1.4E-05	-4.4	Cd244	CD244 natural killer cell receptor 2B4
3.5E-06	-4.4	AU023871	expressed sequence AU023871
3.1E-04	-4.4	Bex6	brain expressed gene 6
1.2E-04	-4.3	Ly6a	lymphocyte antigen 6 complex, locus A
9.3E-03	-4.3	Gm11545	predicted gene 11545
5.1E-07	-4.2	Npas4	neuronal PAS domain protein 4
1.9E-03	-4.2	C530008M17Rik	RIKEN cDNA C530008M17 gene
1.1E-06	-4.2	Adam19	a disintegrin and metallopeptidase domain 19 (meltrin beta)
3.1E-07	-4.2	Tesc	tescalcin
2.0E-06	-4.2	Itga2b	integrin alpha 2b
7.1E-07	-4.2	Spint1	serine protease inhibitor, Kunitz type 1
3.5E-04	-4.1	Kif26b	kinesin family member 26B
5.2E-04	-4.1	Gm1564	predicted gene 1564
1.5E-02	-4.1	Ms4a4b	membrane-spanning 4-domains, subfamily A, member 4B

3.3E-04	-4.1	Penk	preproenkephalin
2.7E-08	-4.1	Prg2	proteoglycan 2, bone marrow
8.8E-06	-4.1	Spata13	spermatogenesis associated 13
2.5E-06	-4.0	Col18a1	collagen, type XVIII, alpha 1
1.8E-03	-3.9	Lyzl4	lysozyme-like 4
2.6E-07	-3.9	Alox12	arachidonate 12-lipoxygenase
2.8E-05	-3.8	Syn3	synapsin III
2.1E-06	-3.8	Rab27b	RAB27b, member RAS oncogene family
3.0E-07	-3.7	Ahrr	aryl-hydrocarbon receptor repressor
3.2E-03	-3.7	St8sia1	ST8 alpha-N-acetyl-neuraminide alpha-2,8-sialyltransferase 1
4.1E-03	-3.7	Cspg4	chondroitin sulfate proteoglycan 4
1.9E-05	-3.7	Mmrn1	multimerin 1
6.1E-06	-3.7	Ciita	class II transactivator
1.0E-04	-3.7	Clec4b1	C-type lectin domain family 4, member b1
6.6E-05	-3.6	Grap2	GRB2-related adaptor protein 2
4.8E-03	-3.6	Mapk13	mitogen-activated protein kinase 13
5.8E-04	-3.6	Fcrls	Fc receptor-like S, scavenger receptor
1.9E-02	-3.6	Ypel4	yippee-like 4 (Drosophila)
9.5E-04	-3.6	Ocstamp	osteoclast stimulatory transmembrane protein
6.7E-03	-3.6	Gm16034	predicted gene 16034
7.2E-07	-3.5	Ngp	neutrophilic granule protein
1.5E-06	-3.5	Mefv	Mediterranean fever
8.7E-06	-3.5	Clec4a1	C-type lectin domain family 4, member a1
7.3E-03	-3.5	Gcm2	glial cells missing homolog 2 (Drosophila)
1.3E-05	-3.4	Ccr7	chemokine (C-C motif) receptor 7
1.5E-02	-3.4	4930562C15Rik	RIKEN cDNA 4930562C15 gene
1.0E-02	-3.3	Syt13	synaptotagmin XIII
1.1E-06	-3.3	Gnaz	guanine nucleotide binding protein, alpha z subunit
1.6E-07	-3.3	Angptl2	angiopoietin-like 2
1.2E-04	-3.2	Fhl1	four and a half LIM domains 1
1.1E-05	-3.2	H2-Ab1	histocompatibility 2, class II antigen A, beta 1
2.4E-03	-3.2	Rhcg	Rhesus blood group-associated C glycoprotein
9.7E-03	-3.2	Slc36a2	solute carrier family 36 (proton/amino acid symporter), member 2
2.8E-06	-3.2	Ache	acetylcholinesterase
1.1E-05	-3.2	Siglech	sialic acid binding Ig-like lectin H
2.9E-04	-3.2	Ch25h	cholesterol 25-hydroxylase
3.7E-05	-3.2	H2-Aa	histocompatibility 2, class II antigen A, alpha
3.6E-06	-3.2	Diras2	DIRAS family, GTP-binding RAS-like 2
1.2E-07	-3.1	Rnd3	Rho family GTPase 3
2.8E-05	-3.1	H2-Eb2	histocompatibility 2, class II antigen E beta2

4.3E-07	-3.1	Rasgrp4	RAS guanyl releasing protein 4
1.0E-06	-3.1	Egr3	early growth response 3
9.1E-03	-3.1	Klrb1c	killer cell lectin-like receptor subfamily B member 1C
1.3E-05	-3.1	Cd74	CD74 antigen (invariant polypeptide of major histocompatibility complex, class II antigen-associated)
6.1E-03	-3.1	Gm13520	predicted gene 13520
1.5E-07	-3.1	Clec4a2	C-type lectin domain family 4, member a2
7.9E-05	-3.1	Alox5	arachidonate 5-lipoxygenase
1.1E-04	-3.0	Nhedc2	Na+/H+ exchanger domain containing 2
2.7E-05	-3.0	Kalrn	kalirin, RhoGEF kinase
1.2E-05	-3.0	L1cam	L1 cell adhesion molecule
4.4E-05	-3.0	Cyp2s1	cytochrome P450, family 2, subfamily s, polypeptide 1
1.2E-05	-3.0	Pdgfb	platelet derived growth factor, B polypeptide
1.9E-03	-3.0	I830012O16Rik	RIKEN cDNA I830012O16 gene
4.1E-07	-3.0	Gfi1b	growth factor independent 1B
4.1E-06	-3.0	Fos	FBJ osteosarcoma oncogene
1.1E-02	-3.0	Alox15	arachidonate 15-lipoxygenase
9.3E-07	-3.0	Vcan	versican
1.4E-03	-3.0	Gprc5c	G protein-coupled receptor, family C, group 5, member C
5.0E-06	-3.0	Vipr1	vasoactive intestinal peptide receptor 1
1.6E-04	-3.0	Tubb1	tubulin, beta 1 class VI
2.6E-03	-3.0	Mctp2	multiple C2 domains, transmembrane 2
3.0E-06	-2.9	Il10	interleukin 10
5.3E-05	-2.9	Clec4a4	C-type lectin domain family 4, member a4
7.8E-06	-2.9	Gm11771	predicted gene 11771
1.9E-05	-2.9	H2-Eb1	histocompatibility 2, class II antigen E beta
1.2E-08	-2.9	Lipn	lipase, family member N
4.1E-03	-2.9	Gm10604	predicted gene 10604
8.8E-03	-2.9	Snord89	small nucleolar RNA, C/D box 89
4.8E-03	-2.9	Dscam	Down syndrome cell adhesion molecule
5.9E-03	-2.9	Gm8369	predicted gene 8369
5.1E-04	-2.9	Efr3b	EFR3 homolog B (S. cerevisiae)
5.6E-03	-2.9	Fhod3	formin homology 2 domain containing 3
1.5E-03	-2.9	Nap1l2	nucleosome assembly protein 1-like 2
2.4E-05	-2.8	Pald1	phosphatase domain containing, paladin 1
2.6E-07	-2.8	Mmp8	matrix metallopeptidase 8
1.1E-06	-2.8	Tgfbi	transforming growth factor, beta induced
2.0E-04	-2.8	Oas3	2'-5' oligoadenylate synthetase 3
1.8E-07	-2.8	Serpine2	serine (or cysteine) peptidase inhibitor, clade E, member 2
1.7E-05	-2.8	Prr5l	proline rich 5 like

6.0E-04	-2.8	Tshz3	teashirt zinc finger family member 3
4.9E-04	-2.8	Ms4a4c	membrane-spanning 4-domains, subfamily A, member 4C
9.5E-05	-2.7	Samd14	sterile alpha motif domain containing 14
1.4E-07	-2.7	Plec	plectin
2.2E-05	-2.7	Egfr	epidermal growth factor receptor
4.9E-05	-2.6	Scimp	SLP adaptor and CSK interacting membrane protein
5.4E-07	-2.6	Ms4a7	membrane-spanning 4-domains, subfamily A, member 7
4.2E-05	-2.6	Prkcq	protein kinase C, theta
1.1E-03	-2.6	Tas1r3	taste receptor, type 1, member 3
2.4E-03	-2.6	Flrt3	fibronectin leucine rich transmembrane protein 3
3.5E-06	-2.6	Mmp13	matrix metallopeptidase 13
1.2E-05	-2.6	Gpr34	G protein-coupled receptor 34
6.0E-03	-2.6	Slc13a3	solute carrier family 13 (sodium-dependent dicarboxylate transporter), member 3
1.8E-06	-2.6	Zfyve28	zinc finger, FYVE domain containing 28
2.2E-05	-2.6	Ramp3	receptor (calcitonin) activity modifying protein 3
3.3E-03	-2.5	Kcne3	potassium voltage-gated channel, Isk-related subfamily, gene 3
1.2E-08	-2.5	Lmna	lamin A
1.1E-05	-2.5	B3gnt5	UDP-GlcNAc:betaGal beta-1,3-N-acetylglucosaminyltransferase 5
2.3E-06	-2.5	Timp3	tissue inhibitor of metalloproteinase 3
4.4E-07	-2.5	Havcr2	hepatitis A virus cellular receptor 2
5.8E-03	-2.5	Ifit1	interferon-induced protein with tetratricopeptide repeats 1
1.6E-05	-2.5	Acap1	ArfGAP with coiled-coil, ankyrin repeat and PH domains 1
1.9E-02	-2.5	Apol9a	apolipoprotein L 9a
1.5E-03	-2.5	Fgf13	fibroblast growth factor 13
1.6E-02	-2.5	Gm11760	predicted gene 11760
1.8E-05	-2.4	Apol7c	apolipoprotein L 7c
8.2E-07	-2.4	Nr4a1	nuclear receptor subfamily 4, group A, member 1
1.4E-03	-2.4	Gm15156	predicted gene 15156
6.7E-05	-2.4	Cbr2	carbonyl reductase 2
7.5E-06	-2.4	Gp9	glycoprotein 9 (platelet)
2.2E-03	-2.4	Ifit3	interferon-induced protein with tetratricopeptide repeats 3
7.8E-05	-2.4	4931403E22Rik	RIKEN cDNA 4931403E22 gene
1.8E-03	-2.4	Hpx	hemopexin
7.8E-06	-2.4	Angptl4	angiopoietin-like 4
1.3E-05	-2.4	Cd300lg	CD300 antigen like family member G

1.6E-04	-2.4	Efna1	ephrin A1
4.0E-03	-2.4	Mir27b	microRNA 27b
2.8E-06	-2.4	Il18rap	interleukin 18 receptor accessory protein
3.8E-03	-2.3	Wnt9a	wingless-type MMTV integration site 9A
1.6E-03	-2.3	Tcte2	t-complex-associated testis expressed 2
2.8E-05	-2.3	Fbln5	fibulin 5
2.7E-04	-2.3	Elovl7	ELOVL family member 7, elongation of long chain fatty acids (yeast)
3.8E-03	-2.3	Kcnk12	potassium channel, subfamily K, member 12
1.6E-03	-2.3	Dgkg	diacylglycerol kinase, gamma
1.1E-03	-2.3	Sez6l2	seizure related 6 homolog like 2
3.1E-05	-2.3	Hpgd	hydroxyprostaglandin dehydrogenase 15 (NAD)
5.7E-05	-2.3	Cx3cr1	chemokine (C-X3-C) receptor 1
1.6E-02	-2.3	Gm8428	predicted gene 8428
1.4E-06	-2.3	Ms4a6d	membrane-spanning 4-domains, subfamily A, member 6D
2.7E-06	-2.3	Treml1	triggering receptor expressed on myeloid cells-like 1
8.4E-05	-2.3	Gm12291	predicted gene 12291
6.8E-03	-2.3	Npdc1	neural proliferation, differentiation and control gene 1
4.9E-07	-2.3	Trib1	tribbles homolog 1 (Drosophila)
3.0E-05	-2.2	Skint3	selection and upkeep of intraepithelial T cells 3
2.4E-06	-2.2	Cd72	CD72 antigen
3.5E-07	-2.2	Gp1bb	glycoprotein Ib, beta polypeptide
1.1E-03	-2.2	C430049B03Rik	RIKEN cDNA C430049B03 gene
2.2E-06	-2.2	Mfsd2b	major facilitator superfamily domain containing 2B
2.9E-05	-2.2	Galnt9	UDP-N-acetyl-alpha-D-galactosamine:polypeptide N-acetylgalactosaminyltransferase 9
2.6E-09	-2.2	2010002N04Rik	RIKEN cDNA 2010002N04 gene
2.7E-04	-2.2	Ptrf	polymerase I and transcript release factor
6.8E-05	-2.2	Spnb1	spectrin beta 1
2.5E-04	-2.2	Ctla2a	cytotoxic T lymphocyte-associated protein 2 alpha
1.9E-05	-2.2	Ms4a6b	membrane-spanning 4-domains, subfamily A, member 6B
1.0E-03	-2.2	Pcdh7	protocadherin 7
1.2E-02	-2.1	Oasl2	2'-5' oligoadenylate synthetase-like 2
3.3E-05	-2.1	Ceacam19	carcinoembryonic antigen-related cell adhesion molecule 19
8.3E-07	-2.1	Tiam1	T cell lymphoma invasion and metastasis 1
4.9E-04	-2.1	Ahr	aryl-hydrocarbon receptor
8.1E-04	-2.1	Nlrp1c	NLR family, pyrin domain containing 1C
5.0E-05	-2.1	Ndrg4	N-myc downstream regulated gene 4

2.6E-05	-2.1	Ndrg1	N-myc downstream regulated gene 1
1.2E-08	-2.1	Vim	vimentin
5.5E-03	-2.1	Ppic	peptidylprolyl isomerase C
3.6E-06	-2.1	Pdpn	podoplanin
3.1E-06	-2.1	4831426I19Rik	RIKEN cDNA 4831426I19 gene
4.2E-06	-2.1	Ms4a6c	membrane-spanning 4-domains, subfamily A, member 6C
7.6E-03	-2.1	Adora3	adenosine A3 receptor
8.5E-04	-2.1	Cd5l	CD5 antigen-like
3.2E-03	-2.1	Rhbdf1	rhomboid family 1 (Drosophila)
5.2E-04	-2.1	Oas2	2'-5' oligoadenylate synthetase 2
2.7E-06	-2.1	Olfm1	olfactomedin 1
2.1E-08	-2.1	Clec4n	C-type lectin domain family 4, member n
8.4E-05	-2.1	Clec4a3	C-type lectin domain family 4, member a3
8.5E-06	-2.1	Adcy6	adenylate cyclase 6
1.4E-03	-2.1	Fgfr1	fibroblast growth factor receptor 1
9.2E-03	-2.1	Hoxa1	homeobox A1
1.3E-06	-2.1	Gpr56	G protein-coupled receptor 56
2.8E-04	-2.1	Boc	biregional cell adhesion molecule-related/ down-regulated by oncogenes (Cdon) binding protein
1.3E-04	-2.1	Rhoj	ras homolog gene family, member J
7.8E-03	-2.1	Cnih3	cornichon homolog 3 (Drosophila)
1.3E-09	-2.1	Capn2	calpain 2
6.3E-04	-2.0	Prune2	prune homolog 2 (Drosophila)
2.4E-04	-2.0	Mmp14	matrix metallopeptidase 14 (membrane-inserted)
8.2E-07	-2.0	Cd109	CD109 antigen
9.3E-04	-2.0	Cdk5r1	cyclin-dependent kinase 5, regulatory subunit 1 (p35)
7.7E-04	-2.0	Pvrl1	poliovirus receptor-related 1
4.0E-03	-2.0	Gm13966	predicted gene 13966
7.3E-07	-2.0	S100a4	S100 calcium binding protein A4
1.8E-05	-2.0	Gpr141	G protein-coupled receptor 141
2.6E-03	-2.0	Cacnb3	calcium channel, voltage-dependent, beta 3 subunit
1.6E-07	-2.0	Cytip	cytohesin 1 interacting protein
3.5E-05	-2.0	Itgal	integrin alpha L
6.1E-04	-2.0	Nckap1	NCK-associated protein 1
7.9E-07	-2.0	Dusp1	dual specificity phosphatase 1
5.1E-07	-2.0	Itgb3	integrin beta 3
1.8E-04	-2.0	Gm10693	predicted pseudogene 10693
9.7E-03	-2.0	Gm11950	predicted gene 11950
1.2E-02	-2.0	Gpr68	G protein-coupled receptor 68
1.9E-07	-2.0	S100a6	S100 calcium binding protein A6 (calcyclin)
5.4E-03	-2.0	Gm17035	predicted gene 17035

2.7E-04	-2.0	Eya4	eyes absent 4 homolog (Drosophila)
7.4E-08	-2.0	Cdc42ep3	CDC42 effector protein (Rho GTPase binding) 3
8.8E-08	-1.9	Tnip3	TNFAIP3 interacting protein 3
4.6E-04	-1.9	B430305J03Rik	RIKEN cDNA B430305J03 gene
1.7E-06	-1.9	Dpep2	dipeptidase 2
2.5E-03	-1.9	Irf7	interferon regulatory factor 7
2.0E-05	-1.9	Nedd4	neural precursor cell expressed, developmentally down-regulated 4
4.0E-05	-1.9	Car5b	carbonic anhydrase 5b, mitochondrial
6.9E-05	-1.9	Mcoln3	mucolipin 3
1.5E-04	-1.9	H2-DMa	histocompatibility 2, class II, locus DMa
2.0E-06	-1.9	Fscn1	fascin homolog 1, actin bundling protein (Strongylocentrotus purpuratus)
1.9E-05	-1.9	Emp2	epithelial membrane protein 2
3.3E-08	-1.9	Lifr	leukemia inhibitory factor receptor
8.6E-05	-1.9	Emr4	EGF-like module containing, mucin-like, hormone receptor-like sequence 4
2.1E-05	-1.9	Gm14005	predicted gene 14005
2.8E-06	-1.9	Gp1ba	glycoprotein 1b, alpha polypeptide
2.9E-08	-1.9	Gm14548	predicted gene 14548
3.3E-06	-1.9	Zranb3	zinc finger, RAN-binding domain containing 3
4.4E-03	-1.9	Rac3	RAS-related C3 botulinum substrate 3
4.6E-05	-1.9	Napsa	napsin A aspartic peptidase
1.2E-03	-1.9	Pira2	paired-Ig-like receptor A2
1.1E-04	-1.9	Myom1	myomesin 1
4.1E-06	-1.9	Spsb1	splA/ryanodine receptor domain and SOCS box containing 1
3.3E-03	-1.9	Cacna1b	calcium channel, voltage-dependent, N type, alpha 1B subunit
4.6E-06	-1.9	Naaa	N-acylethanolamine acid amidase
5.6E-03	-1.9	Prr15	proline rich 15
1.8E-05	-1.9	Gpr85	G protein-coupled receptor 85
2.8E-03	-1.9	Rtp4	receptor transporter protein 4
1.6E-02	-1.9	March3	membrane-associated ring finger (C3HC4) 3
8.3E-03	-1.9	Ugt1a6a	UDP glucuronosyltransferase 1 family, polypeptide A6A
8.7E-06	-1.8	Entpd1	ectonucleoside triphosphate diphosphohydrolase 1
6.8E-06	-1.8	Lilra6	leukocyte immunoglobulin-like receptor, subfamily A (with TM domain), member 6
5.3E-08	-1.8	S100a10	S100 calcium binding protein A10 (calpactin)
3.1E-03	-1.8	Myo1d	myosin ID
9.0E-04	-1.8	Gm12951	predicted gene 12951
1.3E-02	-1.8	Pcdhb21	protocadherin beta 21

6.7E-06	-1.8	Fgf11	fibroblast growth factor 11
4.3E-07	-1.8	Myo6	myosin VI
1.0E-06	-1.8	Aim1	absent in melanoma 1
5.3E-03	-1.8	Lipg	lipase, endothelial
8.9E-04	-1.8	Ahnak	AHNAK nucleoprotein (desmoyokin)
1.4E-04	-1.8	Gdpd5	glycerophosphodiester phosphodiesterase domain containing 5
1.1E-06	-1.8	Fam84b	family with sequence similarity 84, member B
4.1E-04	-1.8	Pigz	phosphatidylinositol glycan anchor biosynthesis, class Z
7.3E-03	-1.8	Tmem190	transmembrane protein 190
1.9E-07	-1.8	Ccrn4l	CCR4 carbon catabolite repression 4-like (S. cerevisiae)
6.4E-06	-1.8	Thra	thyroid hormone receptor alpha
2.0E-03	-1.8	Ccr3	chemokine (C-C motif) receptor 3
1.3E-04	-1.8	P2ry14	purinergic receptor P2Y, G-protein coupled, 14
1.8E-07	-1.8	Irf2bpl	interferon regulatory factor 2 binding protein-like
9.2E-04	-1.8	Rpl31-ps18	ribosomal protein L31, pseudogene 18
8.8E-06	-1.8	Kdr	kinase insert domain protein receptor
1.1E-02	-1.8	AA474408	expressed sequence AA474408
5.0E-03	-1.8	Mamld1	mastermind-like domain containing 1
7.9E-05	-1.8	Casp4	caspase 4, apoptosis-related cysteine peptidase
3.8E-03	-1.8	Dner	delta/notch-like EGF-related receptor
1.5E-06	-1.8	Fxyd2	FXYD domain-containing ion transport regulator 2
3.2E-05	-1.8	Clec5a	C-type lectin domain family 5, member a
4.1E-03	-1.8	Nr1d1	nuclear receptor subfamily 1, group D, member 1
5.0E-03	-1.8	Cmpk2	cytidine monophosphate (UMP-CMP) kinase 2, mitochondrial
1.7E-03	-1.8	Gm15922	predicted gene 15922
1.0E-02	-1.7	Kif5a	kinesin family member 5A
5.3E-03	-1.7	Hspa12b	heat shock protein 12B
1.1E-05	-1.7	Ass1	argininosuccinate synthetase 1
4.3E-05	-1.7	Lsr	lipolysis stimulated lipoprotein receptor
1.4E-05	-1.7	Mgst3	microsomal glutathione S-transferase 3
1.6E-03	-1.7	AI427809	expressed sequence AI427809
2.0E-06	-1.7	Sult1a1	sulfotransferase family 1A, phenol-preferring, member 1
3.0E-05	-1.7	Map3k6	mitogen-activated protein kinase kinase kinase 6
3.4E-03	-1.7	Ifi27l2a	interferon, alpha-inducible protein 27 like 2A
3.0E-04	-1.7	Tnfsf8	tumor necrosis factor (ligand) superfamily, member 8
1.5E-03	-1.7	Rpl31-ps1	ribosomal protein L31, pseudogene 1
1.2E-05	-1.7	Frmd6	FERM domain containing 6
3.8E-04	-1.7	Tm6sf1	transmembrane 6 superfamily member 1
2.7E-03	-1.7	Samd9l	sterile alpha motif domain containing 9-like
1.0E-05	-1.7	Gbp9	guanylate-binding protein 9

1.1E-04	-1.7	Prdm1	PR domain containing 1, with ZNF domain
1.9E-02	-1.7	Ank1	ankyrin 1, erythroid
6.1E-04	-1.7	Fkbp1b	FK506 binding protein 1b
1.6E-04	-1.7	Sh2d1b1	SH2 domain protein 1B1
1.2E-02	-1.7	Fut7	fucosyltransferase 7
5.4E-07	-1.7	Spn	sialophorin
1.4E-05	-1.7	Slamf9	SLAM family member 9
1.8E-03	-1.7	Klf8	Kruppel-like factor 8
2.5E-05	-1.7	Arl4c	ADP-ribosylation factor-like 4C
1.4E-05	-1.7	Gm5424	predicted gene 5424
2.7E-07	-1.7	Ctsk	cathepsin K
6.9E-04	-1.7	Slc24a3	solute carrier family 24 (sodium/potassium/calcium exchanger), member 3
2.4E-03	-1.7	Gm10575	predicted gene 10575
2.1E-05	-1.7	Pdlim2	PDZ and LIM domain 2
3.3E-05	-1.7	Met	met proto-oncogene
1.2E-06	-1.7	Fgd3	FYVE, RhoGEF and PH domain containing 3
3.1E-05	-1.7	Cd4	CD4 antigen
1.3E-02	-1.7	Ak7	adenylate kinase 7
8.9E-05	-1.7	Nqo1	NAD(P)H dehydrogenase, quinone 1
1.6E-04	-1.7	Hist1h2bp	histone cluster 1, H2bp
1.3E-02	-1.7	Gm10357	predicted gene 10357
3.1E-04	-1.7	Gm15931	predicted gene 15931
7.3E-04	-1.7	Fnbp1l	formin binding protein 1-like
2.5E-09	-1.6	Emp1	epithelial membrane protein 1
3.3E-05	-1.6	Bmf	BCL2 modifying factor
9.5E-04	-1.6	Stbd1	starch binding domain 1
8.4E-05	-1.6	Niacr1	niacin receptor 1
1.4E-06	-1.6	Calhm2	calcium homeostasis modulator 2
6.4E-08	-1.6	Gsn	gelsolin
2.4E-04	-1.6	H2-DMb2	histocompatibility 2, class II, locus Mb2
1.7E-02	-1.6	Fah	fumarylacetoacetate hydrolase
9.7E-04	-1.6	Trim2	tripartite motif-containing 2
2.1E-03	-1.6	Slfn5	schlafen 5
3.9E-06	-1.6	Ube2l6	ubiquitin-conjugating enzyme E2L 6
1.5E-02	-1.6	Gm7609	predicted pseudogene 7609
4.5E-05	-1.6	Mfap3l	microfibrillar-associated protein 3-like
1.7E-04	-1.6	Hist1h2bk	histone cluster 1, H2bk
2.1E-06	-1.6	Cxcl16	chemokine (C-X-C motif) ligand 16
7.9E-06	-1.6	Gpr183	G protein-coupled receptor 183
1.8E-04	-1.6	Rnase4	ribonuclease, RNase A family 4
2.9E-03	-1.6	Stap1	signal transducing adaptor family member 1

2.9E-09	-1.6	Anxa2	annexin A2
2.9E-04	-1.6	Arhgap27	Rho GTPase activating protein 27
3.7E-05	-1.6	N4bp3	NEDD4 binding protein 3
6.7E-07	-1.6	Mfsd6	major facilitator superfamily domain containing 6
8.2E-06	-1.6	Anxa1	annexin A1
1.5E-06	-1.6	Susd3	sushi domain containing 3
2.7E-07	-1.6	Tsc22d3	TSC22 domain family, member 3
4.2E-05	-1.6	Gm11709	predicted gene 11709
8.7E-05	-1.6	Mical2	microtubule associated monoxygenase, calponin and LIM domain containing 2
1.9E-06	-1.6	Myo1g	myosin IG
2.0E-07	-1.6	Camkk2	calcium/calmodulin-dependent protein kinase kinase 2, beta
1.9E-02	-1.6	Tsku	tsukushi
2.3E-04	-1.6	Slc22a15	solute carrier family 22 (organic anion/cation transporter), member 15
3.4E-05	-1.6	Gm11710	predicted gene 11710
1.6E-05	-1.6	Mocos	molybdenum cofactor sulfurase
1.9E-05	-1.6	Mllt4	myeloid/lymphoid or mixed-lineage leukemia (trithorax homolog, Drosophila); translocated to, 4
1.2E-06	-1.6	Oit3	oncoprotein induced transcript 3
4.5E-08	-1.6	Esyt1	extended synaptotagmin-like protein 1
1.7E-03	-1.6	Tmem37	transmembrane protein 37
1.3E-04	-1.6	Ang	angiogenin, ribonuclease, RNase A family, 5
1.7E-02	-1.6	9830001H06Rik	RIKEN cDNA 9830001H06 gene
3.6E-04	-1.6	Uchl1	ubiquitin carboxy-terminal hydrolase L1
1.9E-07	-1.6	Fam105a	family with sequence similarity 105, member A
1.1E-06	-1.6	Gcnt1	glucosaminyl (N-acetyl) transferase 1, core 2
8.8E-05	-1.6	Maml3	mastermind like 3 (Drosophila)
5.9E-05	-1.6	Cd300lh	CD300 antigen like family member H
2.2E-05	-1.6	Plxdc1	plexin domain containing 1
9.3E-04	-1.6	2610034B18Rik	RIKEN cDNA 2610034B18 gene
3.7E-04	-1.6	Gnb4	guanine nucleotide binding protein (G protein), beta 4
8.6E-03	-1.6	Gm7582	predicted gene 7582
1.1E-06	-1.6	Sema6d	sema domain, transmembrane domain (TM), and cytoplasmic domain, (semaphorin) 6D
2.3E-03	-1.6	Ramp1	receptor (calcitonin) activity modifying protein 1
6.8E-03	-1.6	Gm15930	predicted gene 15930
7.8E-05	-1.6	Ophn1	oligophrenin 1
4.5E-05	-1.5	Gm11711	predicted gene 11711
1.1E-05	-1.5	Icam1	intercellular adhesion molecule 1
9.0E-03	-1.5	Hist1h2bn	histone cluster 1, H2bn

1.6E-07	-1.5	Fam129b	family with sequence similarity 129, member B
3.4E-04	-1.5	Oas1g	2'-5' oligoadenylate synthetase 1G
2.2E-06	-1.5	Lpl	lipoprotein lipase
1.5E-02	-1.5	Fam151a	family with sequence simliarity 151, member A
1.1E-07	-1.5	Coro1a	coronin, actin binding protein 1A
1.9E-03	-1.5	Tle1	transducin-like enhancer of split 1, homolog of Drosophila E(spl)
1.7E-04	-1.5	Sncaip	synuclein, alpha interacting protein (synphilin)
3.7E-06	-1.5	Smcr7	Smith-Magenis syndrome chromosome region, candidate 7 homolog (human)
1.2E-05	-1.5	Fcgr1	Fc receptor, IgG, high affinity I
7.5E-05	-1.5	Dfna5	deafness, autosomal dominant 5 (human)
1.9E-06	-1.5	Gm5431	predicted gene 5431
8.6E-05	-1.5	Emr1	EGF-like module containing, mucin-like, hormone receptor-like sequence 1
1.3E-02	-1.5	Gpr176	G protein-coupled receptor 176
1.1E-03	-1.5	Dock4	dedicator of cytokinesis 4
1.0E-06	-1.5	Plp2	proteolipid protein 2
1.9E-07	-1.5	Myadm	myeloid-associated differentiation marker
4.0E-06	-1.5	Irgm2	immunity-related GTPase family M member 2
5.3E-06	-1.5	Kcnj2	potassium inwardly-rectifying channel, subfamily J, member 2
1.5E-08	-1.5	Il1rn	interleukin 1 receptor antagonist
4.2E-06	-1.5	Il4i1	interleukin 4 induced 1
5.3E-06	-1.5	Gm6169	predicted gene 6169
2.7E-04	-1.5	Gm13669	predicted gene 13669
9.3E-06	-1.5	Nfam1	Nfat activating molecule with ITAM motif 1
4.5E-05	-1.5	Pik3ap1	phosphoinositide-3-kinase adaptor protein 1
5.7E-03	-1.5	Gm15518	predicted gene 15518
1.3E-07	-1.5	Kcnn4	potassium intermediate/small conductance calcium-activated channel, subfamily N, member 4
1.7E-02	-1.5	Spata25	spermatogenesis associated 25
1.8E-02	-1.5	Gm15448	predicted gene 15448
1.2E-06	-1.5	Rapgef5	Rap guanine nucleotide exchange factor (GEF) 5
1.2E-05	-1.5	Tmem178	transmembrane protein 178
2.0E-04	-1.5	Dhx58	DEXH (Asp-Glu-X-His) box polypeptide 58
9.3E-07	-1.5	Slc46a3	solute carrier family 46, member 3

Supplementary Table S3. List of the 30 most strongly enriched Gene Ontology Biological process terms among the 357 transcripts that were upregulated in the 48h DHR-treated BMDMs compared to the DMSO-treated ones. False Discovery Rate value was calculated by Benjamini-Hochberg procedure for multiple test correction (FDR < 0.05). Strength: Log10(observed gene count / expected gene count).

term ID	term description	observed gene count	background gene count	strength	FDR
GO:0010749	Regulation of nitric oxide mediated signal transduction	3	11	1.28	4.6E-02
GO:0048799	Animal organ maturation	4	22	1.1	2.8E-02
GO:0030201	Heparan sulfate proteoglycan metabolic process	4	25	1.05	3.8E-02
GO:0034104	Negative regulation of tissue remodeling	4	27	1.02	4.6E-02
GO:0045780	Positive regulation of bone resorption	4	27	1.02	4.6E-02
GO:0033198	Response to atp	5	35	1	1.7E-02
GO:1900745	Positive regulation of p38mapk cascade	4	28	1	5.0E-02
GO:0045124	Regulation of bone resorption	7	52	0.97	2.4E-03
GO:0048873	Homeostasis of number of cells within a tissue	5	38	0.96	2.2E-02
GO:0060122	Inner ear receptor cell stereocilium organization	5	40	0.94	2.6E-02
GO:0043277	Apoptotic cell clearance	5	42	0.92	3.0E-02
GO:0007266	Rho protein signal transduction	6	54	0.89	1.5E-02
GO:0090303	Positive regulation of wound healing	6	56	0.88	1.7E-02
GO:1903036	Positive regulation of response to wounding	7	74	0.82	1.2E-02
GO:0034103	Regulation of tissue remodeling	8	87	0.81	6.2E-03
GO:0060563	Neuroepithelial cell differentiation	6	65	0.81	3.0E-02
GO:1990869	Cellular response to chemokine	7	82	0.78	1.9E-02
GO:0060976	Coronary vasculature development	6	71	0.77	4.1E-02
GO:0070098	Chemokine-mediated signaling pathway	6	71	0.77	4.1E-02
GO:0001657	Ureteric bud development	8	97	0.76	1.1E-02
GO:0030595	Leukocyte chemotaxis	10	125	0.75	2.7E-03
GO:0019229	Regulation of vasoconstriction	6	75	0.75	4.9E-02
GO:0006909	Phagocytosis	11	144	0.73	1.9E-03
GO:0072073	Kidney epithelium development	11	146	0.72	2.0E-03
GO:0060993	Kidney morphogenesis	7	93	0.72	3.1E-02
GO:0008360	Regulation of cell shape	11	151	0.71	2.5E-03
GO:0097529	Myeloid leukocyte migration	8	113	0.7	2.2E-02
GO:0002690	Positive regulation of leukocyte chemotaxis	7	97	0.7	3.8E-02

GO:0120162	Positive regulation of cold-induced thermogenesis	7	97	0.7	3.8E-02
GO:2000106	Regulation of leukocyte apoptotic process	7	98	0.7	3.9E-02

Supplementary Table S4. List of 102 differently expressed transcripts between 2h DMSO- or 2h DHR (1 μM)-treated 4 day-differentiated monocytes (based on at least 1.5-fold change and corrected p value<0.05).

**Corr. p val.**	**FC**	**Gene symbol**	**Gene title**
1.8E-08	39.4	Hic1	hypermethylated in cancer 1
1.2E-06	30.4	Camkk1	calcium/calmodulin-dependent protein kinase kinase 1, alpha
3.2E-06	9.7	Art2a-ps	ADP-ribosyltransferase 2a, pseudogene
8.9E-08	8.8	Bmp2	bone morphogenetic protein 2
7.1E-06	6.9	Rarb	retinoic acid receptor, beta
2.1E-05	6.3	B230378P21Rik	RIKEN cDNA B230378P21 gene
1.6E-03	5.2	Fam20a	family with sequence similarity 20, member A
2.3E-05	4.9	Gm11464	predicted gene 11464
1.4E-02	3.8	Cxcl9	chemokine (C-X-C motif) ligand 9
4.3E-05	3.8	Kcnip3	Kv channel interacting protein 3, calsenilin
1.4E-03	3.7	Kcnf1	potassium voltage-gated channel, subfamily F, member 1
9.1E-07	3.7	Vegfa	vascular endothelial growth factor A
1.0E-02	3.5	Pcdhb16	protocadherin beta 16
4.9E-08	3.4	Pram1	PML-RAR alpha-regulated adaptor molecule 1
6.1E-03	3.4	AI848285	expressed sequence AI848285
8.6E-05	3.3	Hbegf	heparin-binding EGF-like growth factor
2.1E-07	3.3	Hivep2	human immunodeficiency virus type I enhancer binding protein 2
3.5E-04	3.2	Kif26b	kinesin family member 26B
3.4E-03	3.0	Rpsa-ps3	ribosomal protein SA, pseudogene 3
5.2E-03	3.0	Wnk2	WNK lysine deficient protein kinase 2
1.0E-07	3.0	Smad3	SMAD family member 3
2.8E-05	2.9	Tox3	TOX high mobility group box family member 3
1.2E-03	2.9	AI593442	expressed sequence AI593442
2.9E-04	2.9	Il2rb	interleukin 2 receptor, beta chain
8.3E-03	2.8	Rab30	RAB30, member RAS oncogene family
1.4E-02	2.7	Gm10430	predicted gene 10430
6.9E-07	2.7	Il21r	interleukin 21 receptor
3.6E-03	2.7	Pou3f1	POU domain, class 3, transcription factor 1
1.3E-07	2.6	Rab20	RAB20, member RAS oncogene family
1.3E-10	2.5	Ptgs1	prostaglandin-endoperoxide synthase 1

1.9E-04	2.5	5031425F14Rik	RIKEN cDNA 5031425F14 gene
5.8E-03	2.4	SNORA40	Small nucleolar RNA SNORA40
8.8E-09	2.4	Dtx4	deltex 4 homolog (Drosophila)
2.6E-09	2.3	2510009E07Rik	RIKEN cDNA 2510009E07 gene
6.5E-08	2.3	Osgin1	oxidative stress induced growth inhibitor 1
8.3E-04	2.3	Tubb3	tubulin, beta 3 class III
1.3E-02	2.2	Gm4784	predicted gene 4784
1.4E-04	2.2	Mex3b	mex3 homolog B (C. elegans)
1.6E-07	2.2	Cd38	CD38 antigen
1.1E-09	2.2	Mcart1	mitochondrial carrier triple repeat 1
6.0E-05	2.1	Hist1h3a	histone cluster 1, H3a
4.5E-08	2.1	Fam117a	family with sequence similarity 117, member A
2.0E-07	2.0	Gm13431	predicted gene 13431
1.4E-05	2.0	Hrh1	histamine receptor H1
2.0E-06	2.0	Socs2	suppressor of cytokine signaling 2
6.4E-06	2.0	Ddc	dopa decarboxylase
6.9E-08	2.0	Neurl3	neuralized homolog 3 homolog (Drosophila)
2.5E-06	2.0	Gm22	predicted gene 22
3.1E-04	2.0	Gpr182	G protein-coupled receptor 182
9.8E-07	2.0	Klhl12	kelch-like 12 (Drosophila)
5.9E-05	1.9	Shcbp1l	Shc SH2-domain binding protein 1-like
6.8E-07	1.9	Gadd45a	growth arrest and DNA-damage-inducible 45 alpha
1.3E-06	1.9	BC005764	cDNA sequence BC005764
3.6E-04	1.9	Gdf15	growth differentiation factor 15
1.3E-05	1.9	AA467197	expressed sequence AA467197
1.0E-02	1.9	Kbtbd11	kelch repeat and BTB (POZ) domain containing 11
8.1E-04	1.9	Hmcn1	hemicentin 1
1.2E-05	1.9	Enc1	ectodermal-neural cortex 1
2.5E-07	1.9	Mafb	v-maf musculoaponeurotic fibrosarcoma oncogene family, protein B (avian)
5.4E-07	1.8	Gda	guanine deaminase
8.7E-04	1.8	Xpnpep2	X-prolyl aminopeptidase (aminopeptidase P) 2, membrane-bound
2.1E-06	1.8	Dchs1	dachsous 1 (Drosophila)
3.9E-04	1.8	Vdr	vitamin D receptor
1.6E-03	1.7	AI427809	expressed sequence AI427809
9.7E-03	1.7	E230016M11Rik	RIKEN cDNA E230016M11 gene
1.8E-08	1.7	Dusp5	dual specificity phosphatase 5
4.9E-08	1.7	Fam20c	family with sequence similarity 20, member C
1.5E-05	1.7	Gm6659	predicted gene 6659
1.4E-07	1.7	Ednrb	endothelin receptor type B
9.4E-08	1.7	Nrip1	nuclear receptor interacting protein 1

8.9E-08	1.7	Tagap	T cell activation Rho GTPase activating protein
1.0E-04	1.7	Tecpr2	tectonin beta-propeller repeat containing 2
2.1E-07	1.6	Ikbke	inhibitor of kappaB kinase epsilon
1.2E-04	1.6	Adap2	ArfGAP with dual PH domains 2
1.2E-04	1.6	Vangl2	vang-like 2 (van gogh, Drosophila)
7.5E-07	1.6	Neil2	nei like 2 (E. coli)
3.9E-09	1.6	Gm20507	predicted gene 20507
2.0E-07	1.6	Bcl3	B cell leukemia/lymphoma 3
4.1E-06	1.6	Irf2bp2	interferon regulatory factor 2 binding protein 2
7.9E-05	1.6	Zfp759	zinc finger protein 759
1.2E-02	1.6	Gm16240	predicted gene 16240
5.3E-05	1.6	Fbxo32	F-box protein 32
1.4E-06	1.6	Cmah	cytidine monophospho-N-acetylneuraminic acid hydroxylase
1.4E-06	1.6	Cd97	CD97 antigen
1.6E-07	1.6	Lfng	LFNG O-fucosylpeptide 3-beta-N-acetylglucosaminyltransferase
3.6E-07	1.6	Plk2	polo-like kinase 2
1.9E-07	1.6	Hcfc2	host cell factor C2
5.2E-06	1.6	Sox4	SRY-box containing gene 4
4.0E-06	1.5	Ccrl2	chemokine (C-C motif) receptor-like 2
2.0E-05	1.5	Arrdc3	arrestin domain containing 3
4.2E-06	1.5	Aifm2	apoptosis-inducing factor, mitochondrion-associated 2
1.8E-06	1.5	Spsb4	splA/ryanodine receptor domain and SOCS box containing 4
2.5E-06	1.5	Ndst1	N-deacetylase/N-sulfotransferase (heparan glucosaminyl) 1
1.6E-05	1.5	B3gnt7	UDP-GlcNAc:betaGal beta-1,3-N-acetylglucosaminyltransferase 7
2.6E-06	1.5	Zfp503	zinc finger protein 503
1.5E-07	1.5	Hs6st1	heparan sulfate 6-O-sulfotransferase 1
2.0E-06	1.5	Ptafr	platelet-activating factor receptor
6.2E-08	1.5	Tgm2	transglutaminase 2, C polypeptide
7.2E-07	1.5	Mt2	metallothionein 2
2.0E-08	1.5	Phf15	PHD finger protein 15
1.7E-05	1.5	Fem1c	fem-1 homolog c (C.elegans)
3.2E-06	1.5	Slco2b1	solute carrier organic anion transporter family, member 2b1
